# Multifunctional Carbon Dots for Electrochemical Capacitors Reviewed: Their Opportunities and Challenges

**DOI:** 10.1002/advs.202508000

**Published:** 2025-09-26

**Authors:** Naiyun Liu, Jingwen Yu, Yaxi Li, Yunliang Liu, Sobia Jabeen, Yuanyuan Cheng, Lei Zhou, Dmitri L. Danilov, Peter H. L. Notten, Haitao Li

**Affiliations:** ^1^ Institute for Energy Research School of Chemistry and Chemical Engineering Jiangsu University Zhenjiang 212013 China; ^2^ School of Energy and Power Engineering Jiangsu University Zhenjiang 212013 China; ^3^ Institute of Energy Technologies Fundamental Electrochemistry (IET‐1) Forschungszentrum Jülich D‐52425 Jülich Germany; ^4^ Department of Electrical Engineering Eindhoven University of Technology Eindhoven 5600 MB The Netherlands; ^5^ Centre for Clean Energy Technology University of Technology Sydney Broadway Sydney NSW 2007 Australia

**Keywords:** carbon dots, double‐layer capacitors, electrochemical capacitors, photoassisted, pseudocapacitors

## Abstract

Electrochemical capacitors (ECs) are promising energy storage devices due to their rapid charge/discharge capability. However, they face limitations in energy density, and certain capacitor materials may exhibit poor conductivity and structural instability. Carbon dots (CDs), characterized by their small size, abundant surface functional groups, and controllable properties, show great potential in enhancing the performance of ECs. This review presents the challenges and opportunities associated with multifunctional CDs in ECs. It begins by summarizing the classification and basic working principles of ECs. Next, it discusses in detail the synthesis methods, fundamental physical and chemical properties, and the electrochemical and photoelectrochemical properties of CDs. Subsequently, it explores the application of CDs as electrode additives and electrolyte additives in ECs, highlighting their unique benefits in improving capacitor performance. Additionally, it examines the innovative use of CDs in photo‐assisted capacitors, revealing insights into capacitor technology derived from the synergistic effects of light and electrochemical energy storage processes. Finally, the current challenges facing the applications for CD‐based ECs are discussed, followed by proposing future research opportunities. This review emphasizes the significant role of multifunctional CDs and their promising potential in advancing EC technology.

## Graphical abstract

Carbon dots (CDs) with unique natures of small size, high specific surface area, and tunable surface functional groups significantly enhance the specific capacitance and cycling stability of electrochemical capacitors (ECs). This review systematically discusses critical challenges and opportunities associated with multifunctional CDs in ECs, aiming to provide deep insights into the design and engineering of advanced CD‐based ECs.

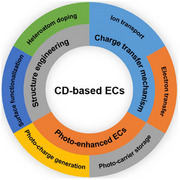

## 1 Introduction

Environmental problems and energy crises are two major global challenges that must be addressed. The effective utilization of renewable energy, as well as improving energy conversion and storage efficiency, is an important measure to avoid energy crises and protect the environment.^[^ ^1^, ^2^ ^]^ Electrochemical energy storage systems, such as electrochemical capacitors (ECs) and batteries, have shown great potential in utilizing new energy, such as solar and wind power.^[^ ^3^, ^4^ ^]^ Among these devices, ECs combine capacitors' fast charging and discharging characteristics with the high storage capacity of batteries. Moreover, ECs present high power densities, superior cycling stability, and good electrochemical reversibility.^[^ ^5^, ^6^, ^7^ ^]^ These advantages make ECs highly applicable in many areas, like portable electronic products, electric vehicles, and backup power supplies. However, their low energy density limits their practical applications. At present, research on ECs has reached a bottleneck. The design and development of high energy‐density ECs with fast (dis)charging and cycling stability have become critical.

Investigating advanced electrode materials with high conductivity and capacitance is vital for developing high‐performance ECs.^[^ ^8^, ^9^, ^10^ ^]^ Promising materials include porous carbon materials (activated carbon, carbon nanofibers, etc.), metal oxides (RuO_2_ and MnO_2_), layered transition metal hydroxides, and MXene.^[^ ^11^, ^12^ ^]^ However, each material has its advantages and disadvantages. For example, activated carbon has a large specific surface area but poor conductivity, while RuO_2_ is expensive and MnO_2_ displays poor electronic conductivity. Layered hydroxides and MXene have suitable ion channels but are prone to stacking, which results in poor cycling stability. Hence, it is essential to tailor the structures of these materials and design new candidates to improve the performance of ECs.

Carbon dots (CDs) are a class of novel 0D carbon nanomaterials known for their remarkable fluorescence properties. They were first reported in 2004 and can be categorized into four main types: carbon quantum dots (CQDs), graphene quantum dots (GQDs), carbon nanodots (CNDs), and carbonized polymer dots (CPDs).^[^ ^13^, ^14^, ^15^ ^]^ In EC applications, CDs stand out among various nanomaterials due to their unique merits of synthesis accessibility, cost efficiency, and structure tunability. These attributes directly address critical limitations of mainstream alternatives like graphene and MXenes. Unlike graphene, which requires energy‐intensive synthesis, or MXenes, which rely on toxic etchants, CDs utilize low‐cost, abundant precursors (including biomass waste and industrial byproducts) and scalable synthesis routes (hydrothermal, pyrolysis, microwave‐assisted methods) to achieve high yields at lower costs. That not only reduces production expenses but also aligns with sustainability goals, overcoming a key barrier to large‐scale fabrication of high‐performance nanomaterials. In addition, CDs possess readily tunable surface chemistry. That is a key advantage over graphene which introduces functional groups via harsh oxidation, resulting in compromised conductivity. CDs intrinsically have rich oxygen‐containing groups (─COOH, ─OH) and can be further functionalized with heteroatoms (N, P, S) or organic moieties (─NH_2_, ─SH) via simple post‐treatment. Overall, in contrast to graphene and MXenes, CDs with synthesis accessibility, cost efficiency, and structure tunability would be one of the most promising candidates applied in ECs.

Such versatility extends beyond ECs to applications in electrocatalysis, photocatalysis, and photovoltaics.^[^ ^16^, ^17^, ^18^, ^19^ ^]^ In ECs specifically, CDs function as conductive bridges in composite electrodes (e.g., CDs/porous carbon^[^ ^20^ ^]^), surface modifiers for pseudocapacitive materials (e.g., CDs/MnO_2_^[^ ^21^ ^]^), and even active redox centers. They maintain structural tunability through doping or functional group modification to optimize electrochemical performance. All these characteristics help address limitations of mainstream materials.^[^ ^22^, ^23^, ^24^ ^]^

**Figure 1:**
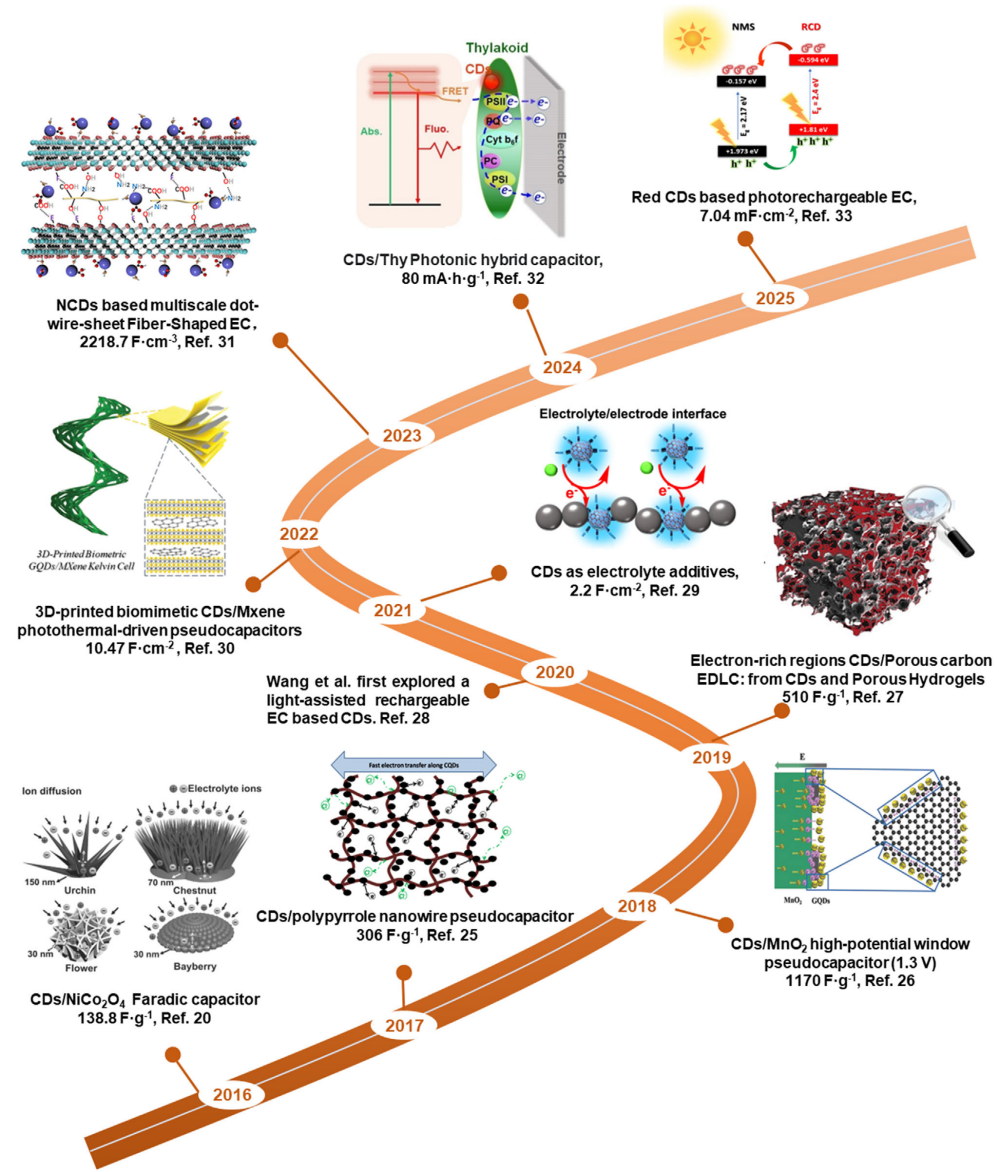
Timeline showing recent advances in the development and utilization of CDs in ECs.^[^ ^20^, ^25^, ^26^, ^27^, ^28^, ^29^, ^30^, ^31^, ^32^, ^33^ ^]^

**Figure** **1** provides a timeline of recent advancements in the development and application of CDs in ECs, underscoring the rapid evolution of this field. Despite these advances, a comprehensive review elucidating the mechanistic roles of CDs in ECs, including electrode engineering, electrolyte optimization, and even photoelectrochemical effects, is still lacking. This review distinguishes itself from existing literature in two key aspects. Unlike prior works that primarily summarize applications of CDs in ECs at a macroscopic level, this review delves into the structure‐performance enhancement mechanisms of CDs, dissecting how their crystallinity, heteroatom doping, and surface functionalization modulate interfacial charge transfer, redox kinetics, and structural stability in EC electrodes. Additionally, while most reviews overlook the photoelectrochemical potential of CDs, this work highlights their unique role in light‐assisted ECs, a nascent field where CDs enable visible‐light‐driven charge generation and separation to enhance energy density. These key focuses address critical gaps in the current literature, offering mechanistic insights into CDs‐mediated optimization of ECs and pioneering discussions on photoactive CDs applications that have not been systematically addressed in prior comprehensive reviews.

This review distinguishes itself from existing literature by offering a comprehensive analysis of how the multifunctionality of CDs can transform the design of ECs. Beginning with an overview of EC fundamentals, we systematically examine the synthesis, structural engineering, and electrochemical/photoelectrochemical properties of CDs. In contrast to prior reviews that primarily treat CDs as electrode additives, we provide an in‐depth discussion of their dual functionality as both electrode modifiers and electrolyte additives, revealing their critical role in optimizing interfacial charge transfer and stability. Furthermore, we highlight a groundbreaking discussion on photoelectrochemically active CDs, which enable light‐enhanced ECs—a nascent field with no dedicated reviews to date. Finally, it proposes the future challenges and opportunities of CD‐based EC materials, providing more insights into the design and engineering of advanced ECs.

## 2 Classification and Characteristics of ECs

ECs are promising electrochemical energy storage devices with unique characteristics, including excellent charging and discharging rates and long cycle life. EC devices typically consist of positive and negative electrodes, electrolytes, and separators. The separator is essential in preventing direct contact between the positive and negative electrodes, which could avoid short circuits. The performance of ECs—specifically, their energy density, power density, and cycle stability—mainly depends on the electrode materials and electrolytes used.

Based on charge‐storage mechanisms, ECs can be categorized into three main types: electric double‐layer capacitors (EDLCs), pseudocapacitors, and faradaic capacitors.^[^ ^34^, ^35^ ^]^ EDLCs store energy through the electrochemical adsorption and desorption of ions, offering advantages like high power density, long cycle life and environmental friendliness. However, they face limitations in energy density and cost. Pseudocapacitors rely on fast, reversible surface faradaic redox reactions, providing higher specific capacitance with materials such as transition metal oxides or conductive polymers. Faradaic capacitors, involving bulk‐phase faradaic processes, possess higher energy density but suffer from slower kinetics. Additionally, ECs can be classified as symmetric, asymmetric, or hybrid based on layout configuration, with hybrid capacitors integrating different charge‐storage mechanisms for enhanced performance.^[^ ^36^, ^37^, ^38^, ^39^ ^]^

Despite their potential, the widespread application of ECs is hampered by inherent limitations across these types. CDs, with their unique combination of nanoscale dimensions (typically 2–10 nm), tunable surface chemistry (abundant oxygen/nitrogen functional groups like ─COOH, ─NH_2_), heteroatom doping capability (N, S, P), and favorable electronic properties (conductive sp^2^ domains), emerge as transformative nanoadditives capable of directly addressing these limitations and reshaping the fundamental characteristics of ECs. This section re‐examines the classification of ECs, including EDLCs, pseudocapacitors, and faradaic capacitors, with a specific focus on elucidating how CDs modulate their core charge storage mechanisms and, in turn, enhance their overall performance attributes.

### 2.1 Charge Storage Mechanism of EDLCs

**Figure 2:**
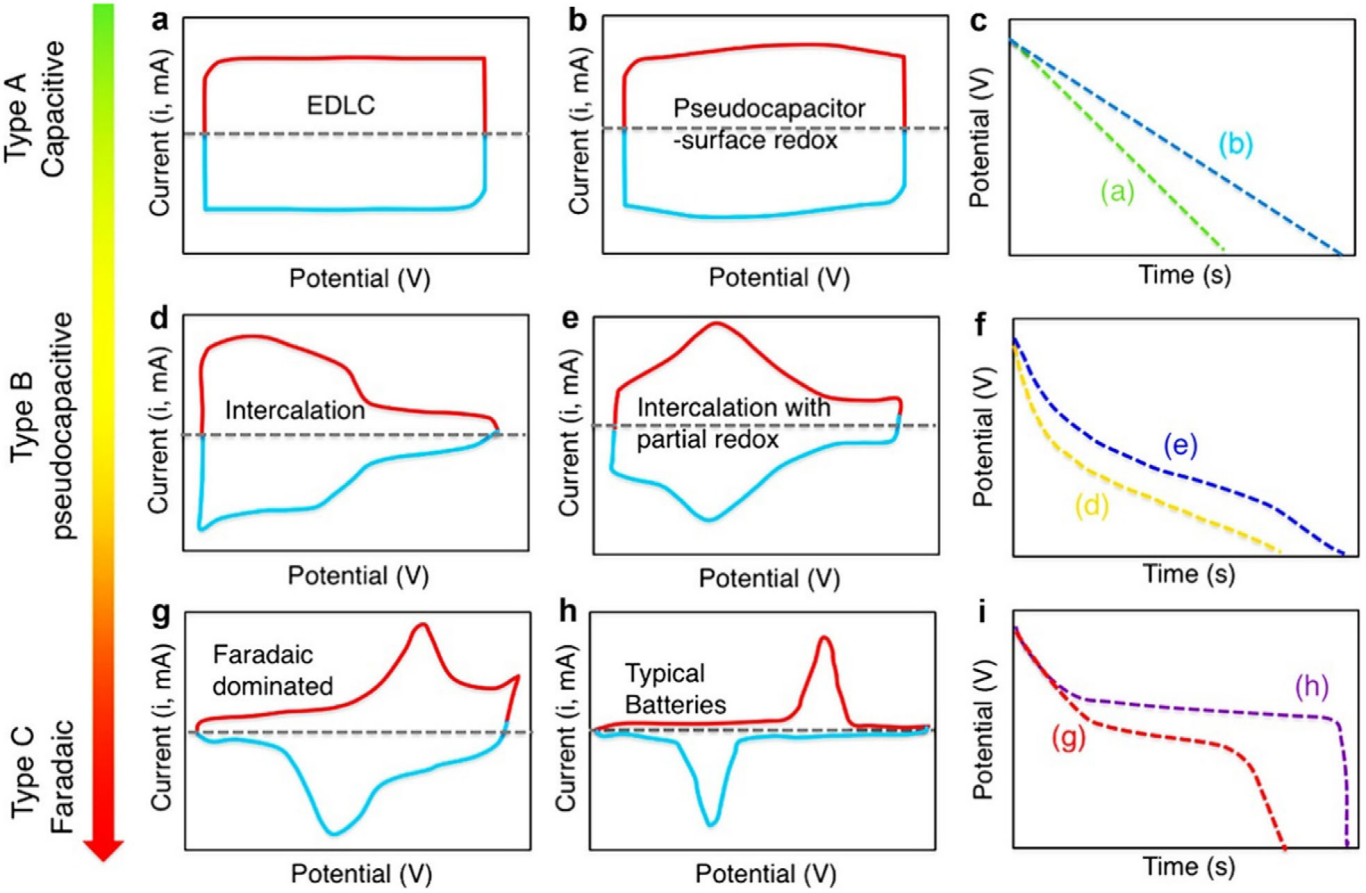
a,b,d,e,g,h) Schematic cyclic voltammograms and c,f,i) corresponding galvanostatic discharge curves for various energy‐storage materials. Reproduced with permission.^[^ ^40^ ^]^ Copyright 2018, American Chemical Society.

EDLCs function by adsorbing anions and cations at the electrode‐electrolyte interface to store and release energy. During charging, electrons flow from the negative to the positive electrode through an external circuit, while cations and anions migrate towards the negative and positive electrodes, respectively, in the electrolyte. This process forms an electrical double‐layer, which can be described by the Helmholtz model, expressed as

(1) $$C=\frac{\varepsilon A}{d}$$

where *C* (F) is a double‐layer capacitance, *ε* (F m^−1^) is the dielectric constant for separating charges, *A* (m^2^) is the electrode surface area, and *d* (m) is the distance between the electrode and electrolyte ions. The discharge process involves the reverse migration of electrons and ions. Notably, the charge–discharge reactions of EDLCs are non‐faradaic and reversible, characterized by rectangular cyclic voltammetry (CV) curves and linear voltage responses during constant‐current charging/discharging (**Figure** **2**a,c). Porous carbon‐based materials, such as activated carbon,^[^ ^41^ ^]^ carbon fiber,^[^ ^42^ ^]^ carbon aerogel,^[^ ^43^ ^]^ carbon nanotubes,^[^ ^44^ ^]^ typically exhibit EDLC‐type behavior. The capacitive performance of the EDLCs depends on the specific surface area and pore structures of electrode materials that allow access to electrolyte ions.

Despite their excellent electrochemical stability, temperature adaptability, and long cycle life, the low energy density of EDLCs restricts their practical applications.^[^ ^45^ ^]^ CDs effectively address these limitations through multiple pathways. Owing to their nanoscale dimensions (2–10 nm), CDs can penetrate and fill the mesopores of traditional carbon electrodes, significantly increasing the electrochemically accessible surface area. For instance, in carbon nanofiber composites, the incorporation of CDs has been reported to boost the specific surface area from 140 m^2^ g^−1^ to over 2000 m^2^ g^−1^, creating hierarchical pore structures that enhance ion diffusion kinetics.^[^ ^46^ ^]^ GQDs, a subtype of CDs with crystalline structures, form conductive percolation networks within amorphous carbon electrodes, reducing the equivalent series resistance (ESR) and accelerating charge transfer. This enhancement in charge transfer kinetics, enabled by the conductive networks formed by CDs, typically leads to a marked improvement in the charging and discharging rates of EDLCs. Multiple studies across various CD‐incorporated electrode systems have reported enhanced capacitive performance.^[^ ^47^ ^]^ In addition, the surface functional groups of CDs improve electrode wettability; for example, ─COOH and ─OH groups form hydrogen bonds with electrolyte ions, reducing layer spacing and constructing electron‐rich regions on the electrode surfaces for absorbing cations, thus boosting capacitance.^[^ ^20^ ^]^

### 2.2 Charge Storage Mechanism of Pseudocapacitors

Conway coined the term “pseudocapacitor” to describe electrode systems that exhibit capacitor‐like electrochemical signatures (e.g., linear voltage‐charge relationships) while relying on fast, surface‐confined faradaic redox reactions rather than purely non‐faradaic charge adsorption in EDLCs.^[^ ^48^ ^]^ “Pseudocapacitors” serve as a bridge between EDLCs and faradaic capacitors. Unlike battery‐type materials, pseudocapacitive materials undergo reversible surface/near‐surface redox reactions with capacitor‐like kinetics (minimal diffusion limitations), resulting in a linear voltage‐time dependence during galvanostatic charge/discharge, similar to EDLC behavior but with higher intrinsic capacitance.

The charge storage mechanism in pseudocapacitive materials mainly involves surface or near‐surface redox reactions (intrinsic pseudocapacitor) and interlayer reactions (intercalation pseudocapacitor). Additionally, when engineered at the nanoscale, certain traditional battery‐type materials exhibit pseudocapacitive properties and are referred to as “extrinsic pseudocapacitor” materials. Compared to EDLC materials, pseudocapacitor electrode materials generally offer higher energy storage capacity. However, the faradaic redox reactions in pseudocapacitors are slower than the non‐faradic processes in EDLCs, leading to lower power density and a shorter cycle life.

#### 2.2.1 Intrinsic Pseudocapacitors

Intrinsic pseudocapacitive materials store charges using rapid surface/near‐surface redox reactions or ion inserting/extracting, with their pseudocapacitive properties remaining unaffected by size. These materials display electrochemical characteristics similar to EDLCs, featuring approximately rectangular CV curves and linear GCD curves (Figure 2b,c). Common materials used in intrinsic pseudocapacitors include transition metal oxide compounds, such as manganese dioxide (MnO_2_), ruthenium dioxide (RuO_2_), and conductive polymers. The redox reactions in these pseudocapacitors occur at the electrode‐electrolyte interface or within the bulk electrodes, contributing to a higher energy density. Key electrochemical features of pseudocapacitance include a linear or pseudo‐linear relationship between the applied potential and the resulting charge, near‐ideal electrochemical reversibility, and surface‐controlled kinetics.

Nanotechnology advancements have led to the development of nanostructured battery‐type materials with reduced ion diffusion distances, mitigating phase changes, and endowing them with electrochemical characteristics similar to pseudocapacitive materials. Dunn and co‐workers termed materials with this characteristic as “extrinsic pseudocapacitive” materials.^[^ ^49^ ^]^ For materials like LiCoO_2_, when the particle size is reduced to less than 10 nm, their electrochemical profile changes from that of a traditional battery‐type to a capacitive profile. Similarly, some researchers have also considered nanosized V_2_O_5_ and other materials as “extrinsic pseudocapacitive” materials.^[^ ^50^, ^51^ ^]^

Transition metal oxides, such as MnO_2_, face challenges like low conductivity and structural degradation during cycling. CDs effectively address these issues. Graphitic CDs, especially GQDs, act as conductive scaffolds within insulating metal oxides. By bridging particles or decorating surfaces, GQDs reduce charge transfer resistance, enabling full utilization of the redox‐active material and maintaining fast kinetics even at high rates.^[^ ^52^ ^]^ The surface functional groups and heteroatom dopants (e.g., ─COOH, ─NH_2_, N/S) in CDs can participate in or catalyze additional faradaic reactions, enhancing the overall capacitance of the device.^[^ ^53^, ^54^ ^]^

#### 2.2.2 Intercalation Pseudocapacitor

Some layered materials, such as TiO_2_(B), Nb_2_O_5_, Ti_3_C_2_, and MoO_3_, can incorporate electrolyte ions (like Li^+^, Na^+^, K^+^, and H^+^) into their tunnels or layers through Faradic charge transfer without undergoing a crystal phase transition.^[^ ^55^ ^]^ This unique ability, known as “intercalation pseudocapacitance,”^[^ ^56^ ^]^ allows for rapid and efficient charge storage and release, making these materials suitable for energy storage applications. The electrochemical curves of typical intercalated pseudocapacitive material Nb_2_O_5_ (Figure 2d) and Ti_3_C_2_ (Figure 2e) material display broad and reversible redox peaks, characterized by a linear relationship between current and scan rate, minimal capacity change with charging time, and negligible changes in peak potential with scan rate. While cation‐intercalated pseudocapacitors share some similarities with lithium‐ion batteries, the key difference lies in the control mechanism: intercalation pseudocapacitors are governed by surface‐controlled behavior, whereas lithium‐ion batteries rely on bulk‐electrode‐controlled diffusion.

Although current research on the application of CDs in intercalation pseudocapacitors is limited, their unique nanostructure and surface properties hold great promise. CDs can potentially act as interlayer spacers, expanding the d‐spacing between layers (e.g., increasing from 1.01 to 1.22 nm in MXenes) and facilitating faster cation diffusion.^[^ ^30^ ^]^ Additionally, the surface functional groups of CDs can interact with electrolyte ions, optimizing the ion‐intercalation and de‐intercalation processes.^[^ ^57^ ^]^ These properties suggest that CDs could play a significant role in improving the ion diffusion path of intercalation materials and enhancing their structural stability.

### 2.3 Charge Storage Mechanism of Faradaic Capacitors

Faradaic capacitors, which often incorporate battery‐type electrodes in hybrid configurations, operate via diffusion‐limited bulk faradaic redox reactions, distinguishing their charge‐storage mechanism significantly from that of EDLCs and pseudocapacitors.^[^ ^58^, ^59^ ^]^ As illustrated in Figure 2g,h, their CV prominently feature faradaic redox peaks. These peaks exhibit a notable voltage gap exceeding 0.1–0.2 V between oxidation and reduction processes, a characteristic attributed to phase transitions during charge storage. The galvanostatic charge–discharge curves of faradaic capacitors, as shown in Figure 2i, display distinct voltage plateaus, indicative of the presence of two different phases during the charge–discharge process. Such electrochemical signatures result from the bulk‐phase faradaic reactions occurring within the electrode materials.

Battery‐type electrodes, the core components of faradaic capacitors, typically rely on materials that include Ni, Co, Cu, Cd oxides/hydroxides, sulfides/selenides, and their phosphates, which react with hydroxide ions in an alkaline medium to store electrical charge.^[^ ^60^, ^61^, ^62^ ^]^ To accurately differentiate the electrochemical behavior of pseudocapacitors and faradaic capacitors, researchers often analyze the peak position in CV curves and the plateaus in galvanostatic charge–discharge curves. A more quantitative approach involves the kinetic analysis using the equation

(2) $$i=av^{b}$$

where *i* represents the peak current (mA), *v* is the scanning rate (mV s^−1^), and *a* and *b* are adjustable parameters. For faradaic processes, the peak current typically varies with the square root of the scanning rate (*b* = 0.5), which suggests semi‐infinite diffusion‐controlled faradaic processes. If the value of *b* falls between 0.5 and 1, the electrode material exhibits both faradaic and pseudocapacitive properties. When *b* is greater than or equal to 1 (*b* ≈ 1), the behavior is considered pseudocapacitive, meaning the peak current *i* increases linearly with the scanning rate *v*, indicating capacitive control.

However, faradaic capacitors are plagued by intrinsic limitations, such as sluggish ion diffusion and structural instability during repeated charge–discharge cycles, which severely hinder their rate capability and cycle life. CDs provide nanoscale solutions to these problems. The conductive sp^2^ domains in CDs create electron transport pathways in poorly conductive metal compound matrices, speeding up electron transfer.^[^ ^63^ ^]^ More importantly, CDs act as nanoconfinement agents, restricting the growth of active material nanoparticles or decorating their surfaces,^[^ ^64^ ^]^ which suppresses phase transitions and buffers volume expansion, improving structural integrity and cycling stability. Heteroatom‐doped CDs (N, S) introduce extra redox‐active sites and modify the host material's electronic structure, potentially increasing capacity.^[^ ^65^ ^]^ Density functional theory (DFT) studies show that CDs can facilitate charge transfer and reorganize electron density at interfaces, refining reaction kinetics.^[^ ^66^ ^]^

Although CD‐enhanced faradaic capacitors still show redox peaks in CV curves and plateaus in GCD curves, their kinetics improve with mixed diffusion‐ and surface‐controlled behavior, and cycle life extends compared to unmodified ones, making them more suitable for high energy density applications in hybrid capacitors.^[^ ^67^ ^]^

In conclusion, CDs have shown the remarkable ability to target the specific limitations of each EC type. In EDLCs, they can optimize porosity and interfacial properties; in pseudocapacitors, they can enhance conductivity, promote redox activity, and stabilize structures; in faradaic capacitors, they can boost conductivity, provide nanoconfinement, and accelerate reaction kinetics. That makes CDs a universal performance enhancer for ECs, paving the way for developing next‐generation ECs with high power, high energy, and long cycle life. The subsequent section explores the synthesis, properties, and experimental validations of CDs within electrode/electrolyte systems.

## 3 Synthesis and Properties of CDs

**Figure 3:**
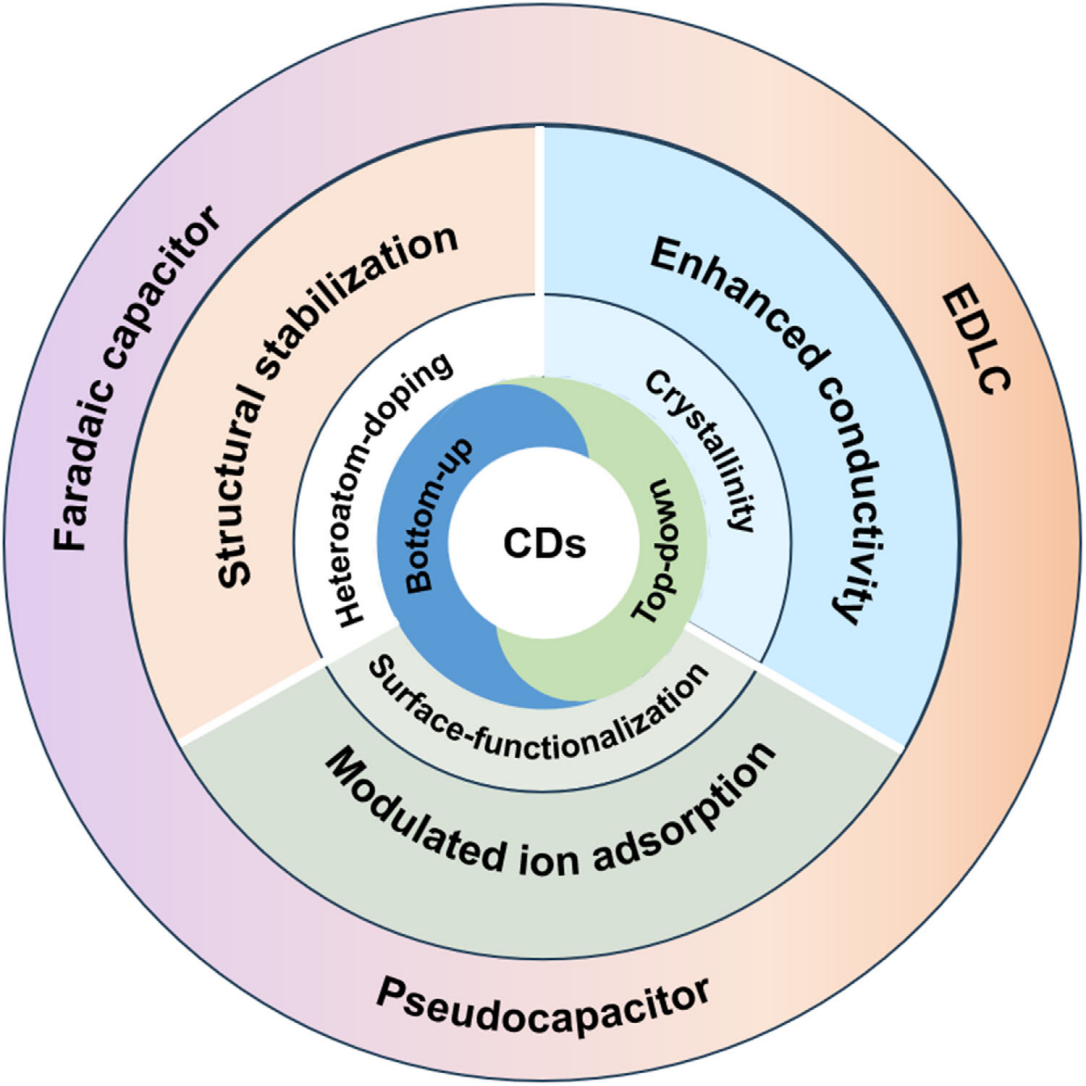
Schematic illustration depicting the synthesis strategies (top‐down and bottom‐up approaches), structural regulation aspects (crystallinity, heteroatom‐doping, surface‐functionalization) of CDs, and their corresponding mechanistic contributions to different types of capacitors, including EDLCs, pseudocapacitors and faradaic capacitors.

CDs have emerged as versatile nanomaterials that address key challenges in ECs, including interfacial resistance, ion diffusion limitations, and structural instability during cycling (**Figure** **3**). Their unique combination of ultrasmall size, tunable surface chemistry, and excellent electrical conductivity enables tailored solutions for different capacitor architectures.^[^ ^15^, ^68^, ^69^, ^70^ ^]^ For instance, the nanoscale dimensions of CDs help prevent pore blockage in EDLCs, while their heteroatom‐doped surfaces significantly enhance redox kinetics in pseudocapacitors.

This chapter explores how CD synthesis (Section 3.1) and structural regulation (Section 3.2), including heteroatom doping and surface functionalization, enable their synergistic integration with EC electrode materials, as demonstrated in the application sections (Section 4). Additionally, the unique photo/electrochemical properties of CDs are discussed, with their potential in light‐assisted ECs highlighted in Section 5.

### 3.1 Synthesis

In recent years, various methods for synthesizing CDs have emerged, categorized as “top‐down” and “bottom‐up” approaches. Different synthesis methods result in CDs with diverse carbon cores and functional group structures.^[^ ^15^, ^71^ ^]^ High‐crystallinity CDs are mostly obtained from larger carbon structures (such as graphite rods, graphene, and carbon nanotubes) through acid oxidation, electrochemical exfoliation, laser ablation, microwave exfoliation, and other treatments.^[^ ^72^, ^73^ ^]^ These methods break down the original bulk carbon materials into nanoparticles, also known as the “top‐down” approach. The “top‐down” synthesis route has the advantages of clear final product structures, facile large‐scale production, and no impurities. The obtained CDs can be directly used after simple filtration and centrifugation. However, controlling the etching process in the “top‐down” approach can be challenging, and the properties of the CDs are primarily controlled by their size and any surface treatments applied afterward.

The amorphous CDs are primarily produced using the “bottom‐up” method. That method involves converting small carbon molecules such as carbohydrates, organic acids, organic amines, or polymer precursors into amorphous CDs through relatively gentle processes like hydrothermal, pyrolysis, ultrasound, and microwave treatment.^[^ ^74^, ^75^, ^76^, ^77^, ^78^, ^79^ ^]^ Due to the diversity of precursor elements, CDs obtained have abundant surface functional groups and heteroatom doping sites.^[^ ^80^ ^]^ In the “bottom‐up” approach, CDs’ chemical structure, composition, and size can be controlled by optimizing the reaction precursors, time, solvents, and temperature. CDs with uniform and structurally controlled properties can be achieved through this method, but the process is relatively complex, and product purification may be challenging.

The distinct properties of CDs from these synthesis routes dictate their optimal applications. Crystalline CDs from top‐down methods, with their high conductivity, are particularly effective for enhancing electron transport in EDLCs. Meanwhile, amorphous CDs from bottom‐up approaches, with their tunable surface chemistry, excel in pseudocapacitive systems where redox reactions dominate.

### 3.2 Structural Regulation

CDs have emerged as highly versatile nanomaterials for electrochemical capacitors. These ultra‐small particles feature multiple defects, accessible edges, and good water solubility, making them promising for ECs. In the charging and discharging process, the small size of CDs allows them to provide more gaps and pathways for charge transfer, thereby improving the rate capability of the capacitors. CDs’ surface is abundant in diverse functional groups, such as hydroxyl, epoxy/ether, carboxyl, and carbonyl, contributing to their water dispersibility and enabling further functionalization and compounding in various applications.^[^ ^81^, ^82^ ^]^ Furthermore, by surface functionalization and heteroatom doping, the physical, chemical, and electronic properties of CDs can be regulated.^[^ ^83^, ^84^ ^]^

#### 3.2.1 Heteroatom Doping

The electronic structure of CDs can be precisely engineered through heteroatom doping, with N doping being the most extensively studied approach.^[^ ^85^, ^86^ ^]^ N incorporation can be achieved through either in situ doping during synthesis or post‐synthetic treatment.^[^ ^87^ ^]^ Various nitrogen‐containing materials, such as ammonium hydroxide, ethylenediamine, tetrabutylammonium perchlorate, and p‐nitroaniline, can be used as nitrogen sources to obtain N‐doped CDs (N‐CDs).^[^ ^88^, ^89^, ^90^, ^91^, ^92^ ^]^ Zhou et al. hydrothermally prepared N‐CDs with o‐phenylenediamine and folic acid as raw materials.^[^ ^31^ ^]^ Lu et al. synthesized N‐CDs via a hydrothermal method with Ketjen black as the C source and ethylenediamine as the N source.^[^ ^93^ ^]^ Some studies have shown that introducing N‐CDs into the electrode materials of ECs exhibits considerable capacitance enhancement. The formed pyrrolic N and pyridinic N were found to destroy the carbon backbone in the CDs, enhancing the electrochemical activity of edge sites, and contributing to the formation of pseudocapacitance. Meanwhile, graphitic N, which replaces carbon atoms, improves conductivity and rate performance.^[^ ^93^ ^]^ N‐CDs not only enhance pseudocapacitive redox reactions (e.g., in MnO_2_ composites) but also improve the wettability of carbon‐based EDLCs, demonstrating their versatility across EC architectures.

Oxygen‐doped^[^ ^94^ ^]^ sulfur‐doped^[^ ^95^ ^]^ and boron‐doped CDs^[^ ^96^ ^]^ have also been explored. Multi‐element co‐doping of CDs can also be utilized based on specific requirements. Combining elements such as N, S, B, or P for doping allows for synergistic effects between heteroatoms. That can enhance the physical, electronic, and electrocatalytic properties of CDs. As a result, codoped CDs were developed with two or more types of heteroatoms. Li et al. produced N, P‐doped CDs (N, P‐CDs) through a one‐step hydrothermal treatment of oxidized graphene (GO) and (NH_4_)_2_HPO_4_. These N, P‐CDs were then compounded with reduced graphene (rGO) to create N, P‐CDs/rGO composite materials. The electrochemical test results demonstrated that the N, P‐CDs/rGO electrode had a high specific capacitance, higher than that of the N, P‐rGO electrode, CDs/rGO electrode, GO electrode, and rGO electrode. Adding CDs helped reduce the aggregation of rGO and increase its specific surface area. Simultaneously, the combined modification of N and P doping enhanced the specific surface area and electronic conductivity of rGO.^[^ ^97^ ^]^

#### 3.2.2 Surface Modification

Surface modification of CDs leverages their abundant oxygen‐containing groups to tailor interfacial properties and electrochemical behavior.^[^ ^98^, ^99^ ^]^ The abundant oxygen‐containing groups (e.g., hydroxyl, carboxyl, and carbonyl) on CDs’ surfaces allow for covalent or non‐covalent functionalization with molecules, polymers, or surfactants. For instance, grafting sulfonic acid groups (─SO_3_H) onto CDs enhances their hydrophilicity and ion‐adsorption capacity, leading to improved double‐layer capacitance. Non‐covalent modifications, such as π–π stacking with graphene or electrostatic interactions with polymers, enable precise control over CDs dispersion and composite structure. Ruiyi et al. designed tryptophan functionalized CDs (Trp‐GCDs) through a one‐step pyrolysis of citric acid and tryptophan.^[^ ^53^ ^]^ When incorporated into a graphene oxide matrix and coordinated with Ru^3+^ ions, these Trp‐GCDs performed dual functions as both structural spacers preventing graphene aggregation and electronic mediators facilitating charge transfer. The resulting 3D RuO_2_‐Trp‐GCDs‐graphene hybrid architecture, featuring atomically dispersed RuO_2_, exhibited remarkable electrochemical properties due to optimized interfacial charge transport and enhanced catalytic activity. This surface engineering approach yielded exceptional capacitive performance, with the modified hybrid demonstrating significantly improved specific capacitance compared to unmodified counterparts, highlighting the transformative potential of targeted CD functionalization in advanced energy storage applications. The Trp‐GCDs served as both structural spacers and electronic mediators when combined with graphene oxide and Ru^3+^ ions, ultimately forming a 3D RuO_2_‐Trp‐GCDs‐graphene hybrid with atomically dispersed RuO_2_. These interfacial modifications significantly improved electronic conductivity and catalytic activity, resulting in an extraordinary capacitance contribution.

#### 3.2.3 Hybridization with Other Materials

Hybridizing CDs with other electroactive materials represents a powerful strategy for synergistically enhancing EC performance. When combined with carbonaceous materials (e.g., activated carbon, graphene, carbon nanotubes), CDs can act as spacers to prevent agglomeration, increase the accessible surface area, and form continuous conductive networks.^[^ ^46^, ^100^ ^]^ In metal oxide‐based electrodes (e.g., MnO_2_, NiCo_2_O_4_), CDs serve as conductive additives and structural stabilizers, improving electron transfer kinetics and mitigating volume changes during cycling.^[^ ^27^, ^101^ ^]^ For instance, the incorporation of CDs into MnO_2_ nanosheets increases the electrode's specific capacitance by providing additional active sites and facilitating ion diffusion. Additionally, CDs can be integrated with conducting polymers (e.g., polyaniline, polypyrrole) through in situ polymerization or solution mixing, leading to composites with enhanced pseudocapacitive behavior.^[^ ^102^ ^]^ The resulting hybrid materials leverage the complementary properties of CDs (e.g., high surface area, functional groups) and host materials, enabling the development of high‐performance ECs with improved energy density, rate capability, and cycling stability.

Notably, these structural engineering strategies, including heteroatom doping, surface modification, and hybridization, are not limited to optimizing electrochemical performance alone; they also lay the groundwork for CDs’ unique photoelectrochemical properties, which are critical for light‐assisted ECs. Heteroatom doping, for instance, introduces defect states that modulate electronic structures; surface modification with hydrophilic or aromatic groups regulates interfacial interactions; and hybridization with semiconductors synergizes light absorption and charge transport. Therefore, these design principles create an intrinsic link between CDs’ structures and their ability to meet the core demands of light‐driven systems, including efficient light harvesting, charge separation, and stable energy storage. This connection is elaborated in detail in the following section on photoelectrochemical properties.

### 3.3 Electrochemical and Photoelectrochemical Properties

Building upon these structural design principles, CDs exhibit two interrelated sets of functional properties: (i) intrinsic electrochemical characteristics stemming from their carbonaceous core and functional groups, and (ii) photoelectrochemical behaviors arising from quantum confinement and defect states—the latter being particularly relevant for light‐assisted ECs.

CDs’ CV diagram is close to mirror symmetry with no distinct peaks, meaning the typical EDLC behavior.^[^ ^103^ ^]^ The interaction between the carbon core structures and functional groups can influence the electron‐transfer properties of CDs. CDs with crystalline carbon cores obtained from precursors, such as graphite, have higher conductivity. Changes in the size of CDs can also affect conductivity. Smaller CDs have more edge sites. The large number of oxygen‐containing functional groups at the edge of the CDs exhibit unique electrocatalytic and photocatalytic properties. In contrast, the over‐introduced functional groups will impede the electron transfer and reduce the conductivity due to the destruction of the sp^2^ structure.^[^ ^104^ ^]^ Heteroatom doping (e.g., B, S, N) further modulates electronic interactions, endowing CDs with tailored catalytic or conductive properties.

**Figure 4:**
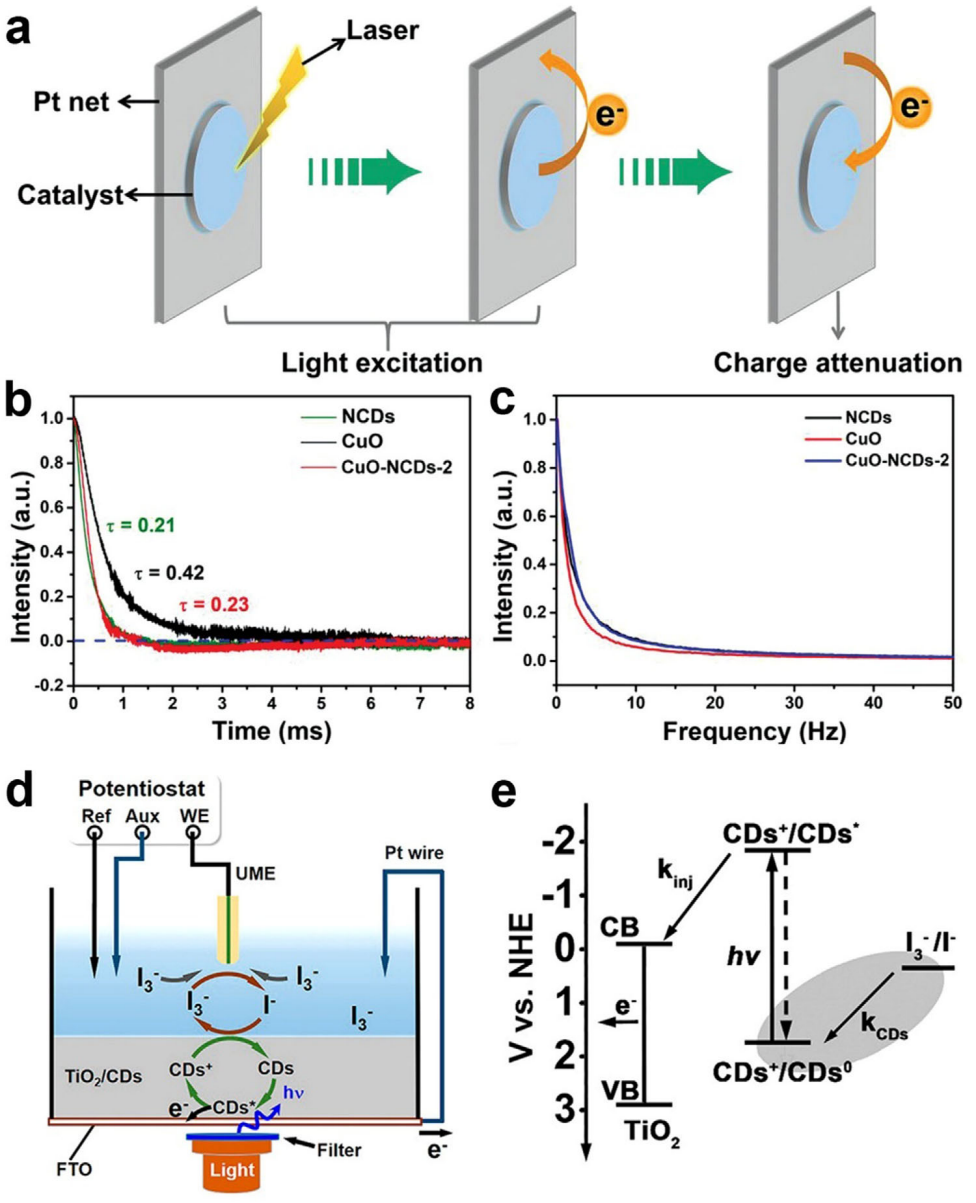
a) Schematic diagram of TPV tests. A 1 cm×1 cm power sample‐coated platinum net functioned as the working electrode, while a platinum wire served as the counter electrode. Excitation of the TPV phenomenon was achieved through a nanosecond laser radiation pulse emitted by a third harmonic Nd:YAG laser (Beamtech Optronics Co., Ltd.). This pulse had a wavelength of 355 nm and a repetition frequency of 5 Hz. The resultant TPV signals underwent amplification via a signal amplifier before being captured by an oscilloscope. b) TPV curves and c) fast Fourier transform curves based on the TPV data of NCDs, CuO, and CuO/NCDs. Reproduced with permission.^[^ ^106^ ^]^ Copyright 2022, WILEY‐VCH. (d) Schematic illustration of scanning electrochemical microscopy setup. (e) Energy levels and electron‐transfer processes of TiO_2_/CDs electrode. Reproduced with permission.^[^ ^107^ ^]^ Copyright 2018, American Chemical Society.

From the perspective of electrochemistry, the conjugated carbon cores of CDs serve as efficient electron transport channels. Recent studies have revealed that CDs exhibit a unique electron sink effect, enabled by their abundant surface functional groups and defect sites. This effect allows CDs to capture, store, and regulate electrons, thereby optimizing electron transport pathways. Kang et al. demonstrated this electron sink behavior using transient photo‐induced voltage (TPV) technology.^[^ ^105^, ^106^ ^]^ TPV technology is characterized by a transient voltage in response to photoelectric stimulation and reveals the detailed process of photogenerated charge transport, storage, and recombination (**Figure** **4**a). The electron decay curve is obtained through TPV testing. Their work showed that the coupling of CDs with Cu/CuO reduced the electron transfer resistance and induced the electron sink, which significantly increased the electron concentration on the catalyst surface (Figure 4b,c).^[^ ^106^ ^]^

Photoelectrochemical properties, critical for light‐assisted ECs, are inherently linked to these structural features: quantum confinement in sp^2^ domains tunes the bandgap (smaller domains with larger bandgaps absorb UV light, larger domains with narrowed bandgaps extend to visible wavelengths), while covalently bound surface functional groups and heteroatom doping further modulate absorption, collectively aligning with solar spectrum requirements. Heteroatom doping introduces defect states that narrow bandgaps and trap charges, thereby reducing recombination and enhancing charge separation. Meanwhile surface functional groups (e.g., ─OH, ─COOH) improve electrolyte wetting and interfacial charge transfer. Aromatic moieties extend π‐conjugation for near‐infrared absorption, and hybridization with semiconductors (e.g., TiO_2_) or electroactive materials (e.g., graphene) synergizes these effects, for example, CDs/TiO_2_ composites show enhanced visible light absorption and accelerated charge transfer kinetics.^[^ ^108^ ^]^ Collectively, these features align CDs' light absorption with the solar spectrum, optimizing their utility in light‐driven systems.

CDs also demonstrate remarkable versatility in charge management, functioning as both electron donors and acceptors to regulate photogenerated carrier dynamics.^[^ ^109^, ^110^ ^]^ This dual functionality has been experimentally verified through fluorescence quenching studies using electron acceptors (2,4‐dinitrotoluene) and electron donors (N, N‐diethylaniline). For example, glucose‐derived CDs rapidly extract electrons from semiconductors to enhance charge separation,^[^ ^77^ ^]^ while CDs in ternary composites act as efficient hole transport channels.^[^ ^81^ ^]^

Advanced characterization techniques have provided deep insights into these charge transfer mechanisms. Liu et al. employed scanning electrochemical microscopy (SECM, Figure 4d) to investigate the reaction kinetics between oxidized CDs and solution‐phase redox pairs in CDs/TiO_2_ composite (Figure 4e).^[^ ^107^ ^]^ Gruebele et al. revealed that photogenerated electrons could migrate from the bulk core to local surface defects in CDs.^[^ ^111^ ^]^ Liu et al. characterized the electron trapping and release dynamics in Ni_2_P/CDs systems using TPV measurements.^[^ ^112^ ^]^ These properties enable practical applications, as seen in PM‐CD composites where CDs facilitate visible light absorption and efficient electron transfer for electrocatalysis,^[^ ^113^ ^]^ highlighting their potential for solar energy conversion and storage technologies.

## 4 Applications of CDs in ECs

The performance of ECs strongly depends on the electrode materials used. Pure CDs tend to polymerize and are rarely used in ECs. Therefore, CDs are frequently used in conjunction with other materials to enhance capacitive performance through their synergistic effects. Additionally, CDs are effective additive materials in aqueous electrolytes for enhancing wettability and ion conductivity, thus contributing to improved capacitive performance. Building on the structural and functional insights from Section 3, CDs are applied as electrode additives and electrolyte modifiers to address the distinct challenges of different EC types. In EDLCs (Section 4.1), their nanoscale size and conductivity optimize pore structure; in pseudocapacitors (Section 4.2), surface groups and heteroatoms enhance redox kinetics; and in faradaic systems (Section 4.3), they mitigate volume changes. These applications highlight CDs as a universal solution for next‐generation ECs.

### 4.1 CDs as Electrode Additive

#### 4.1.1 CDs in Carbon‐Based EDLCs

Carbon nanomaterials, such as activated carbon, carbon fibers, graphene, and carbon nanotubes exhibiting EDLC behavior, are extensively applied in ECs. That is attributed to their high electrical conductivity, large specific surface area, and good stability. However, the inherent limitations of EDLC storage (relying solely on interfacial charge adsorption) lead to low energy density in carbon‐based materials, significantly hindering their application as high‐performance EC electrodes. Emerging studies show that introducing CDs into carbon‐based nanomaterials can optimize specific surface area, engineer suitable pore structures, and enhance charge transport kinetics.

As the most commonly used EDLC electrodes, activated carbons (ACs) suffer from low conductivity and sluggish ion diffusion due to their amorphous microporous structure, restricting capacitance and rate performance.^[^ ^41^ ^]^ To address these limitations, Qing et al. demonstrated that embedding highly crystalline GQDs into AC matrices could establish 3D conductive networks while preserving the microporous framework.^[^ ^114^ ^]^ The resulting composite exhibited an exceptional specific surface area of 2829 m^2^ g^−1^ and achieved a specific capacitance of 388 F g^−1^ at 1 A g^−1^, Notably, ultrahigh energy densities of 13.47 and 7.99 Wh kg^−1^ at power densities of 125 and 12500 W kg^−1^ in alkaline electrolyte. These values significantly outperform commercial AC‐based EDLCs (e.g., SAMWHATM 3000F), which typically exhibit capacitances of 200–250 F g^−1^ in aqueous electrolytes, with energy densities generally below 10 Wh kg^−1^ and power densities around 8000 W kg^−1^. This breakthrough highlights the potential of CDs as functional modifiers to bridge the gap between high energy storage and rapid charge–discharge performance in next‐generation ECs.

Compared to larger carbon materials like graphene, CDs' nanoscale dispersion characteristics enable more uniform construction of electrode conductive systems. In addition, while conductive GQDs enhance electron transport, doped CDs offer additional advantages in regulating hydrophilicity and ion migration. Yu et al. prepared N‐CDs through microwave‐assisted pyrolysis using citric acid and urea as carbon sources.^[^ ^100^ ^]^ The N‐doping endowed N‐CDs with abundant polar groups, which not only improved the hydrophilicity of AC electrodes to accelerate electrolyte penetration into deep pores but also acted as nano‐templates to optimize pore structure, increasing mesopore fraction while slightly reducing microporosity. This dual modification mitigated ion diffusion hindrance in branched pores, boosting the diffusion coefficient by 40% and driving specific capacitance from 125.8 to 301.7 F g^−1^, further highlighting the versatility of CDs in enhancing AC‐based EDLC performance.

**Figure 5:**
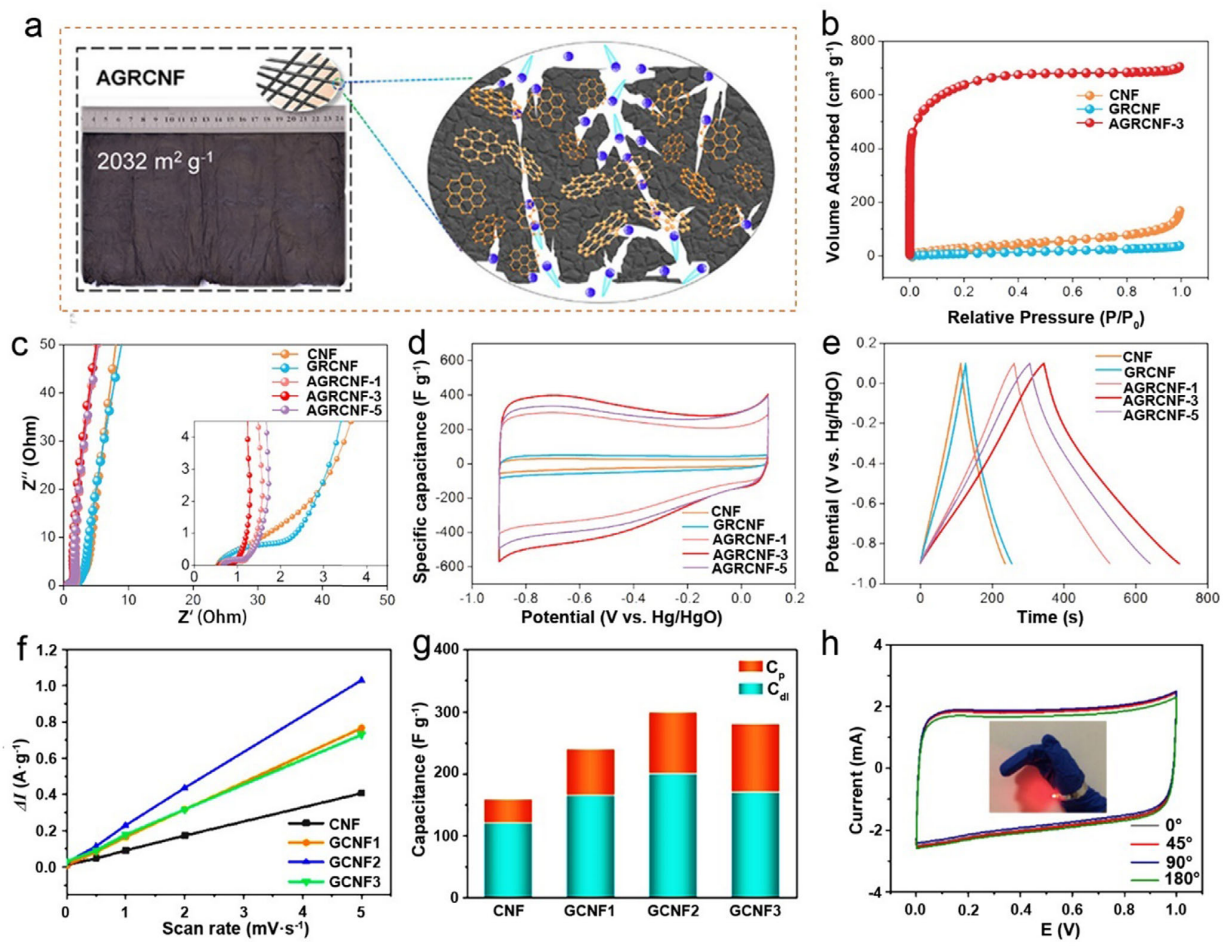
CDs as an additive to CNF electrodes. a) The photo image, b) N_2_ adsorption–desorption isotherms, c) EIS plots, d) CV curves, and e) galvanostatic charge–discharge curves of the electrodes. Reproduced with permission.^[^ ^46^ ^]^ Copyright 2020, American Chemical Society. f) Δ*I* (charge current difference between anode and cathode) versus potential sweep rate curves, g) the total specific capacitance, *C*_dl_, and *C*_p_, h) CV curves of the Flexible ECs using two pieces of CNF/CDs electrodes. Reproduced with permission.^[^ ^115^ ^]^ Copyright 2020, American Chemical Society.

Beyond ACs, carbon nanofiber (CNF) electrodes are also widely used in ECs, especially in flexible ECs, due to their small size, braiding capability, and stable electrochemical properties.^[^ ^116^, ^117^ ^]^ However, their intrinsic performance remains insufficient for practical applications.^[^ ^118^ ^]^ Zhao et al. addressed this by embedding GQDs into CNF fabrics (CNFs/ GQDs, **Figure** **5a**), boosting the specific surface area from 140 to 2032 m^2^ g^−1^ (Figure 5b) and introducing numerous micropores.^[^ ^46^ ^]^ The uniformly embedded GQDs were critical; acting as pore‐forming templates, GQDs created dense microporous structures on and within CNFs (Figure 5c), while π‐π conjugation formed a conductive network, increasing conductivity and reinforcing phase. The composite exhibited rectangular CV curves and isosceles triangular charge‐discharge profiles, characteristic of EDLC behavior (Figure 5d,e) with a specific capacitance (358 F g^−1^ at 1 A g^−1^).

In addition to pore engineering, CDs can also serve as cross‐linkers to enhance structural integrity and introduce pseudocapacitance. Zhang et al. developed a photo‐Fenton method to synthesize GQDs from graphene oxide sheets, fabricating cross‐linked CNF/GQDs composites that integrate enhanced mechanical and electrochemical properties.^[^ ^115^ ^]^ The CV curves of CNF/GQDs deviated from the rectangular shape typical for CNF, indicating the occurrence of redox reactions beyond typical double‐layer capacitance. Using the Dunn model (Figure 5f), Zhang et al. separated double‐layer capacitance (*C*_dl_) and pseudocapacitance (*C*_p_) (Figure 5g). *C*_dl_ first increased then decreased with GQDs content, consistent with the specific surface intruded by the decorated GQDs, while *C*_p_ rose consistently, attributed to oxygen groups enabling pseudocapacitive reactions. The fabricated flexible ECs with two CNF/GQDs electrodes showed a power density of 26 kW kg^−1^, energy density of 6.4 Wh kg^−1^, and nearly the same specific capacitance before and after the bending (Figure 5h).

**Figure 6:**
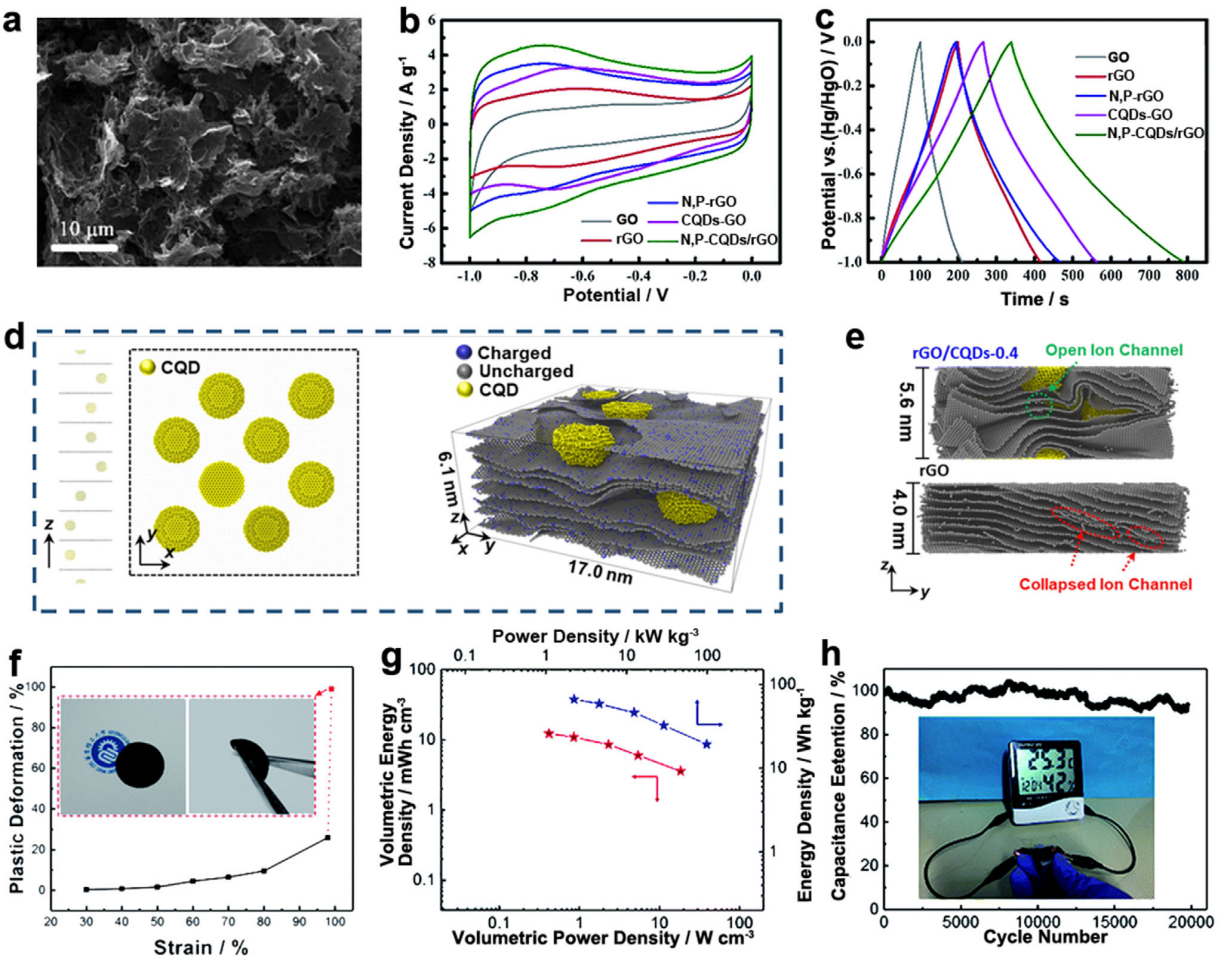
CDs as an additive to Gr electrode. a) FESEM image, b) CV curves at 20 mV s^−1^, and c) GCD curves at 1 A g^−1^ of Gr/CDs electrode. Reproduced with permission.^[^ ^97^ ^]^ Copyright 2019, Royal Society of Chemistry. d) Simulated structure of CDs and CDs/rGO film, and (e) snapshot of the simulated rGO and CDs/rGO film filled with aqueous electrolyte at a compressive strain of 8.0% (360 MPa), with carbon atoms retained to visualize film deformation. Reproduced with permission.^[^ ^119^ ^]^ Copyright 2024, Royal Society of Chemistry. f) Compressive deformation of N‐CDs/Gr framework (inset: photograph at 99.2% strain), g) Ragone plots and h) cycling stability of a capacitor based on two N‐CDs/Gr framework electrodes. Reproduced with permission.^[^ ^120^ ^]^ Copyright 2019, Royal Society of Chemistry.

2D graphene (sp^2^ carbon) has higher conductivity and better crystallinity than usual activated carbon (sp^3^ carbon), enabling rapid electron transfer and electrochemical stability. However, like many 2D materials, graphene nanosheets are prone to stacking during electrode preparation or charging/discharging cycling due to the strong van der Waals force between adjacent layers. That leads to the blockage of electrode micropores and a decrease in specific surface area.^[^ ^47^, ^121^ ^]^ While commercial graphene capacitors reach 200–250 F g^−1^, restacking issues limit their practical capacity. CDs effectively address these limitations through their rich surface functional groups and heteroatom doping, which not only prevent graphene aggregation but also significantly enhance electrochemical performance by introducing additional pseudocapacitive sites and improving charge transfer kinetics.^[^ ^122^ ^]^ Li et al. prepared N, P‐codoped CDs using low‐cost graphite powder as the carbon source and employing (NH_4_)_2_HPO_4_ as both N and P sources through a hydrothermal method.^[^ ^97^ ^]^ When incorporated into rGO aerogel, the resulting N, P‐CDs/rGO presented a unique 3D porous structure where the CDs acted as nanospacers to prevent rGO restacking (**Figure** **6**a). The composite exhibited near‐rectangular CV curves and isosceles triangular charge‐discharge profiles (Figure 6b,c), characteristic of electric double‐layer capacitance, while redox peaks in the CV indicated additional pseudocapacitance from N/P‐containing functional groups and oxygen moieties. This synergistically boosted the overall capacitance to 453.7 F g^−1^, 81–127% higher than commercial EDLC ECs (typically 200–250 F g^−1^). Symmetric devices assembled with N, P‐CDs/rGO aerogels achieved a high energy density of 15.69 Wh kg^−1^ at 325 W kg^−1^, a performance that outperforms commercial graphene‐based Ecs. These typically deliver energy densities in the range of 5–10 Wh kg^−1^ at comparable power densities (300–500 W kg^−1^). For instance, mainstream commercial graphene capacitors (e.g., Maxwell's graphene‐enhanced EDLCs) rarely exceed 8 Wh kg^−1^ in energy density under similar test conditions, highlighting how N, P‐CDs not only mitigate rGO restacking but also synergistically boost energy storage capacity beyond the current graphene capacitor benchmarks.

Building on CD‐modification strategies, Lin et al. employed advanced molecular dynamics simulations to engineer pressure‐tolerant rGO/CDs films.^[^ ^119^ ^]^ The simulations revealed that CDs‐spacers preserved a 0.76 nm interlayer spacing (versus 0.54 nm in pure rGO, Figure 6d), enabling maintained high ion transport coefficient even under 360 MPa pressure (Figure 6e). This structural advantage translated into excellent electrochemical performance: rGO/CDs films retained 81.2% of their initial capacitance (219.7 F cm^−3^) at 360 MPa and maintained a volumetric power density of 59.4 W cm^−3^ at 180 MPa.

In the field of flexible energy storage, Wang et al. developed a compressible N‐doped porous graphene framework (NPGF) by using N‐CDs derived from polyvinyl pyrrolidone (PVP) pyrolysis.^[^ ^120^ ^]^ The N‐CDs served as both a N‐doping source and a structural scaffold. During the microwave‐initiated synthesis, the pyrolysis of PVP generated gaseous byproducts that drove the expansion of graphene sheets, forming a hierarchical porous network with interconnected channels. This process endowed NPGF with outstanding mechanical strength, enabling it to withstand a compressive strain of 99.2% without structural failure (Figure 6f). When assembled into ECs, it yielded a volumetric energy density of 12.3 mWh cm^−3^ at a power density of 0.42 W cm^−3^ (Figure 6g) and retained 90.2% of its capacitance after 20 000 cycles (Figure 6h), highlighting the synergistic enhancement of stability and performance by CDs and graphene.

These studies collectively demonstrate that incorporating CDs into carbon‐based EDLC materials provides multiple optimization effects: it not only enhances double‐layer capacitance by increasing specific surface area and improving electrical conductivity but also introduces pseudocapacitive contributions through abundant surface functional groups. From mechanistic insights, top‐down synthesized CDs (e.g., chemical oxidation method using coal power) with crystalline cores enhance conductivity in AC‐based EDLCs (e.g., 388 F g^−1^ for CDs/AC composites),^[^ ^114^ ^]^ while bottom‐up CDs (e.g., microwave‐assisted method using citric acid and urea) with abundant N‐dopants and N‐functionalities improve electrolyte wettability in AC‐based EDLCs (e.g., 125.8 to 301.7 F g^−1^ with CDs addition). The former excels in electron transport, whereas the latter optimizes ion adsorption, highlighting the trade‐off between conductivity and surface functionality. The synergistic effects lead to superior electrochemical performance while maintaining excellent cycling stability.

#### 4.1.2 CDs in Pseudocapacitors

Compared with EDLCs, pseudocapacitors offer higher energy density but suffer from poor cycle stability due to high resistance during long‐term charge–discharge processes. Recent studies highlight the potential of integrating EDLC and pseudocapacitive materials in a single electrode to enhance energy storage capacity without compromising cycle life or cost. Nanoscale CDs stand out among carbon nanomaterials for their structural flexibility, enabling the fabrication of diverse nanostructures. Their key advantages, including electrochemical conductivity (particularly in GQDs), surface chemistry versatility, and atomic‐scale edge defects, collectively boost the capacitive performance of electrode materials.

These advantages are rooted in three distinct mechanisms: 1) Conductive network formation: CDs with sp^2^‐hybridized carbon cores (e.g., top‐down synthesized GQDs) form percolative networks in metal oxides like RuO_2_, overcoming their intrinsic low conductivity. 2) Interfacial Stabilization: polar functional groups (e.g., ─NH_2_, ─COOH) on CDs establish strong interactions with conductive polymers (e.g., PANI), suppressing volume swelling during cycling. 3) Heteroatom‐driven redox activity: doping CDs with N, S, or P introduces redox‐active sites and modifies electronic structures (e.g., shifting d‐band centers in metal oxides), accelerating faradaic reactions. The following sections detail how these mechanisms enhance the performance of intrinsic (metal oxides, polymers) and intercalation (MXene, MoS_2_) pseudocapacitors.

##### CDs in Intrinsic Pseudocapacitors

Typical intrinsic pseudocapacitors employ transition metal compounds (e.g., RuO_2_, MnO_2_, and Fe_3_O_4_) as electrode materials, but their low conductivity and hydrophilicity limit their applications.^[^ ^53^, ^123^, ^124^, ^125^ ^]^ For RuO_2_ electrodes, Ruiyi et al. developed tryptophan‐functionalized GQDs (Trp‐GQDs) as molecular bridges to construct high‐performance RuO_2_ hybrids, where the Trp‐GQDs served dual critical functions through their unique π‐conjugated structure containing both graphene‐like sheets and benzpyrene groups.^[^ ^53^ ^]^ This molecular design simultaneously enhanced charge transport via π–π stacking interactions with graphene while providing nitrogen coordination sites that strongly bound Ru^3+^ ions, preventing particle aggregation and enabling precise atomic‐level dispersion of RuO_2_. The resulting hybrid material achieved exceptional electrochemical performance with a specific capacitance of 503.7 F g^−1^.

**Figure 7:**
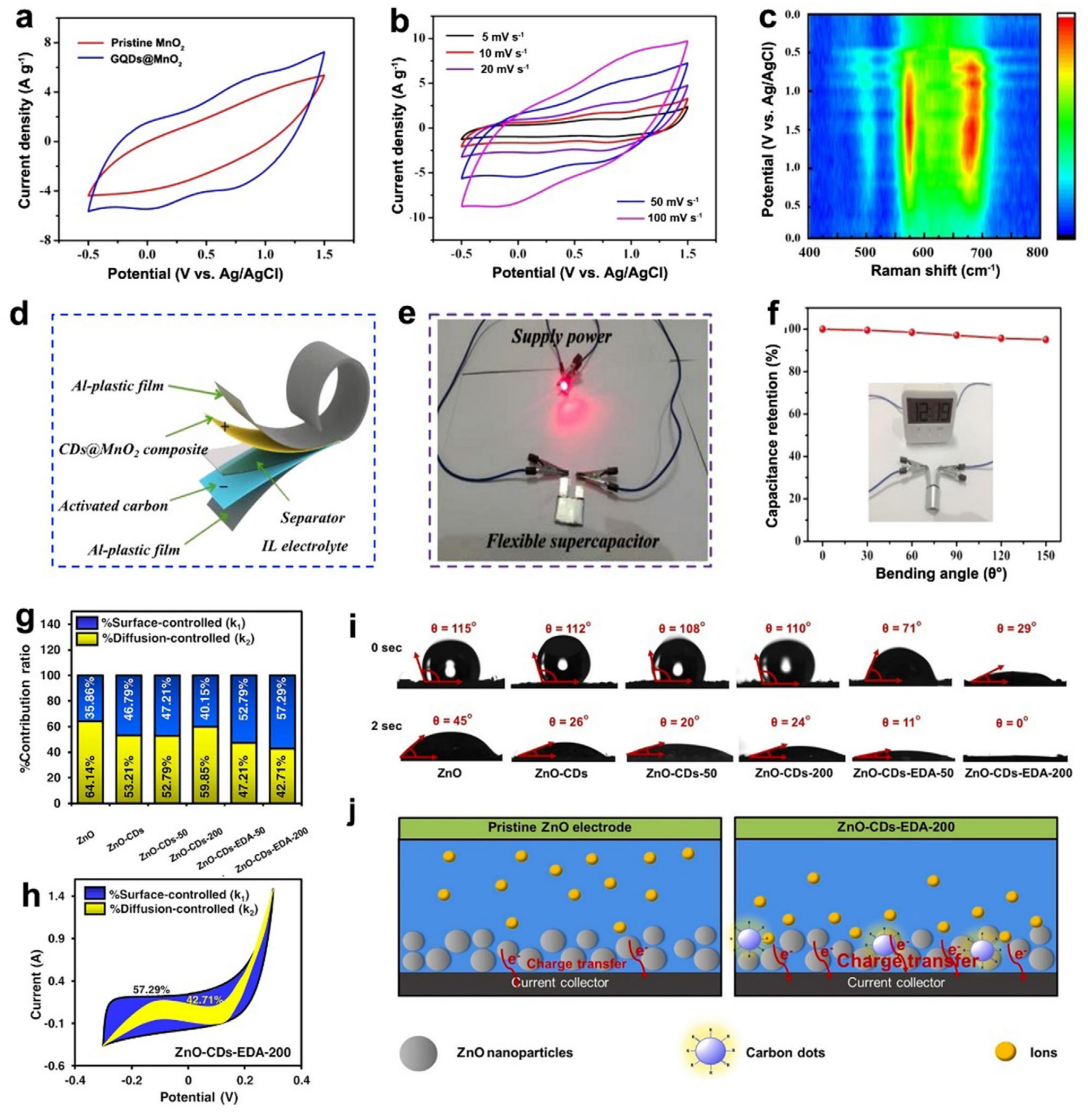
Using CDs as an additive to intrinsic pseudocapacitors. a) CV curves, b) CV profiles scanning at various rates, and c) the corresponding contour map in Raman spectra during the charging and discharging process of MnO_2_/CDs electrode. d) Fabrication procedure, e) the digital photo, and f) the capacitance retention of the flexible device based on MnO_2_/CDs. Reproduced with permission.^[^ ^21^ ^]^ Copyright 2022, Elsevier. g) Contact angles, h) schematic representation of ion/charge transfer process, i) plots, and j) CV curves of the percentage contribution ratio of ZnO/CDs. Reproduced with permission.^[^ ^126^ ^]^ Copyright 2023, Elsevier.

For MnO_2_ electrodes, Zhu et al. synthesized 3D nanoflower‐like GQDs@MnO_2_ composites using conductive GQDs derived via a top‐down graphene oxide process.^[^ ^21^ ^]^ Unlike the molecularly engineered Trp‐GQDs used in RuO_2_ systems that rely on specific π‐conjugation and coordination chemistry, these GQDs primarily functioned as conductivity enhancers through their intrinsic graphene‐like structure, exhibiting an order‐of‐magnitude higher conductivity than pristine MnO_2_. DFT calculations showed GQD incorporation reduced the bandgap from 1.59 to 0.33 eV and enhanced Mn 3d and O 2p electron states, optimizing charge transport. The composite exhibited larger CV integration areas and stable rate‐dependent profiles (**Figure** **7**a,b), with Raman confirming enhanced Mn redox activity (Figure 7c). This yielded a specific capacitance of 208.2 F g^−1^ in organic ionic liquid (IL) electrolytes. Moreover, when used as cathode materials in flexible IL‐based ECs with activated carbon as anode materials (Figure 7d,e), this configuration achieved a high energy density of 82.2 Wh kg^−1^ and a high power density of 11.6 kW kg^−1^, demonstrating good cyclic performance in straight and curved states (Figure 7f).

Phetcharee et al. developed a distinctive approach using gamma‐irradiated ethylenediamine (EDA) to passivate CDs for ZnO‐based ECs.^[^ ^126^ ^]^ Unlike the conductive GQDs used in MnO_2_ systems or the π‐conjugated Trp‐GQDs employed for RuO_2_ hybrids, these gamma‐irradiated amine‐passivated CDs (CDs‐EDA) created through a radiation‐chemical process exhibited unique interfacial properties. Using the Dunn method, the researchers demonstrated that CDs‐EDA significantly enhanced the surface‐controlled processes in ZnO electrodes (Figure 7g), where surface‐controlled capacitance dominated over diffusion‐controlled contributions (Figure 7h). This behavior contrasted with conventional CD‐modification mechanisms, as the gamma‐irradiation process coupled with amine passivation generated abundant amino groups that not only improved electrode wettability (Figure 7i), but also created exceptional charge storage sites at the interface (Figure 7j). Ultimately, the ZnO/CDs electrode displayed a 312% increase in specific capacitance compared to the pure ZnO electrode.

Beyond metal oxide‐based systems, conducting polymers (CPs), such as polyaniline (PANI) and polypyrrole (PPy), have also emerged as promising candidates for intrinsic pseudocapacitive materials.^[^ ^127^, ^128^, ^129^ ^]^ Despite their advantages of high electrical conductivity and ease of fabrication, CPs face the challenge of significant volume changes during cycling, which severely compromises their overall electrochemical performance.^[^ ^8^ ^]^ Many studies have shown that carbonaceous materials can control the physical properties of these polymers by creating percolation networks. The capacitance behavior of the hybrid electrodes is determined by filler size and the interactions between carbonaceous materials and CPs.^[^ ^127^ ^]^ Breczko et al. modified PANI nanotubes with GQDs (PANI/GQDs).^[^ ^130^ ^]^ This composite architecture exhibited superior charge storage characteristics attributable to synergistic effects between its components. The small‐sized GQDs, uniformly dispersed on the PANI nanotube surface, established multiple charge transport pathways through p–π conjugation and hydrogen bonding interactions. Compared to films composed only of PANI nanotubes, the oxidation potential of PANI/GQDs was significantly lowered. The maximum specific capacitance of PANI/GQDs was 245 F g^−1^, which was more than twice that of the original PANI nanotubes. While GQDs enhanced PANI's capacitance by improving charge transfer, N‐CDs offered multifunctional advantages by simultaneously optimizing interfacial interactions and nanostructural stability. Alaş et al. demonstrated this through the synthesis of a ternary Mn‐doped PANI/N‐CDs composite (Mn:PANI:N‐CDs) that exhibited a relatively high specific capacitance.^[^ ^131^ ^]^ The N‐CDs served dual roles: their spherical morphology and surface amine groups maintain PANI's ordered porosity, boosting specific surface area by 21%, while creating ion transport channels. Simultaneously, N‐CDs formed strong interfacial bonds with both the PANI matrix and Mn^2+^ dopants, stabilizing redox‐active sites and facilitating electron transfer. This synergy yielded a high specific capacitance of 595 F g^−1^ and mitigated the structural instability typical of CP electrodes.

##### CDs in Intercalation Pseudocapacitors

Intercalation pseudocapacitor materials, typified by 2D structures like typified by 2D structures like MoS_2_ and MXenes, store charge through cationic intercalation (e.g., Li^+^). These materials nevertheless face critical challenges including limited interlayer active site accessibility, insufficient electrical conductivity, and structural instability during cycling. CD modification has been proven to be particularly effective in addressing these limitations. In MoS_2_‐based systems, the incorporation of N‐GQDs between layers has demonstrated remarkable performance enhancements. Wu et al. prepared N‐GQDs from thiourea and citric acid using a hydrothermal method,^[^ ^132^ ^]^ and reported that N‐GQD intercalation not only expanded the interlayer spacing to expose more active sites but also improved overall electrode wettability through abundant surface functional groups, ultimately achieving an exceptional areal capacitance of 3360 mF cm^−2^. While this intercalation approach successfully addressed the limited active site exposure in pristine MoS_2_, Sharkawy et al. adopted an alternative strategy by constructing flower‐like MoS_2_/N‐CDs composite nanospheres.^[^ ^133^ ^]^ By leveraging the layered structure of MoS_2_ and the excellent electrical conductivity of N‐CDs, the addition of N‐CDs to MoS_2_ enhanced the cyclic stability, wettability, and electrical conductivity of the ECs electrode. The specific capacitance of the electrode reached up to 149.21 F g^−1^ at 0.5 A g^−1^.

**Figure 8:**
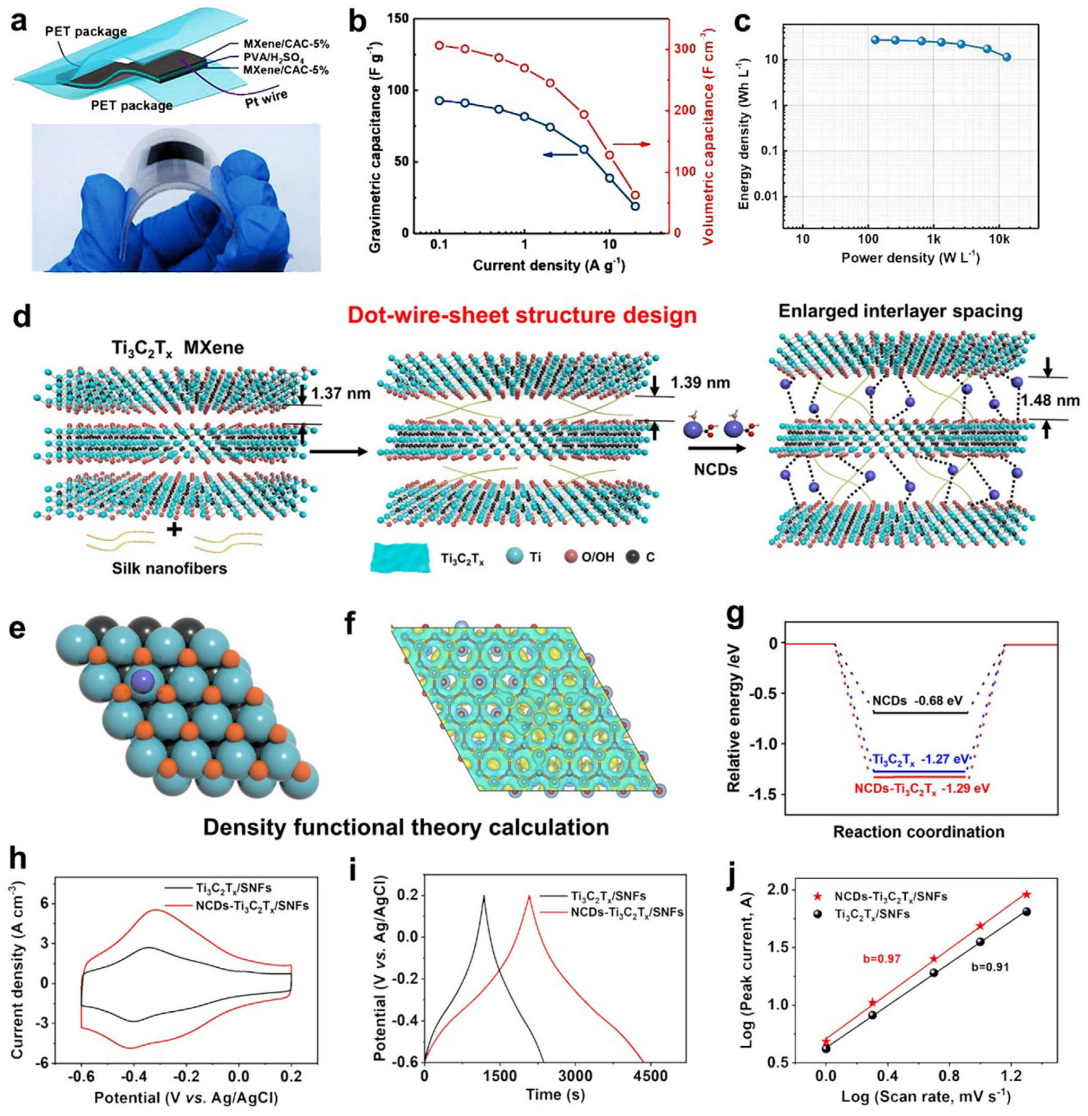
Using CDs as an additive to intercalation pseudocapacitor. a) Schematic illustration and digital photograph, b) CV curves, and c) rate capabilities of MXene/CAC‐based all‐solid‐state symmetric ECs. Reproduced with permission.^[^ ^57^ ^]^ Copyright 2022, WILEY‐VCH. d) Schematic of a multiscale dot‐wire‐sheet structure design concept, e) top view of slab models, f) surface charge difference, g) calculated free energy profiles of H^+^ absorption, h) CV curves, and i) capacitive contributions at different scan rates of NCDs‐Ti_3_C_2_T_x_/SNFs fibers. j) Relationship between capacitance, conduction, and specific surface area with CDs mass loading. Reproduced with permission.^[^ ^31^ ^]^ Copyright 2023, WILEY‐VCH.

MXenes have shown promise as a cationic intercalated 2D material with high electrical conductivity, variable morphology, controllable surface properties, and high‐volume capacitance. However, the strong van der Waals forces between MXene sheets lead to their self‐packing.^[^ ^134^ ^]^ When these sheets are stacked, the pathway for ion transport is lengthened, and the electrolyte's access to the active sites on the surface is reduced, which decreases kinetics and capacitance. Zhang et al. obtained CDs embedded in Ti_3_C_2_T_x_ MXene film (MXene/CAC) electrode material through the carbonization of the gelation of MXene nanosheets with calcium alginate.^[^ ^57^ ^]^ The CDs generated were intercalated within the MXene nanosheets, promoting electrolytic diffusion inside the MXene film and increasing volumetric capacitance, rate capability, and cycling stability. The all‐solid‐state symmetric ECs based on MXene/CAC (**Figure** **8**a) achieved a high gravimetric capacitance of 92.8 F g^−1^, volumetric capacitance of 306.4 F cm^−3^ at 0.1 A g^−1^ (Figure 8b), and a high volumetric energy density of 27.2 Wh L^−1^ (Figure 8c).

In contrast, Zhou et al. developed NCDs‐Ti_3_C_2_T_x_/SNFs hybrid via a more complex multiscale design combining N‐CDs with 1D silk nanofibers (Figure 8d).^[^ ^31^ ^]^ While both studies utilized CDs to address MXene stacking, that approach differed fundamentally by exploiting electronic effects of N‐CDs rather than just physical spacing. As evidenced by DFT calculations, incorporating N‐CDs into the Ti_3_C_2_T_x_ matrix significantly enhanced the delocalization of negative charges across the heterostructure interface. This charge delocalization effect originated from the strong electronic coupling between N‐CDs and Ti_3_C_2_T_x_, creating a more electronegative surface environment. The modified electronic structure provided additional active sites for H^+^ adsorption through strengthened electrostatic interactions while establishing continuous charge transfer pathways that substantially lower the energy barrier for H^+^ ion transport (Figure 8e–g). The integrated CV area of NCDs‐Ti_3_C_2_T_x_/SNFs was larger than that of the sample without N‐CDs (Figure 8h), and the hybrid achieved a remarkable volumetric capacitance of 2218.7 F cm^−3^ in 1 m H_2_SO_4_ electrolyte (Figure 8i). This performance was attributed to CDs‐induced interlayer spacing that facilitated efficient ion diffusion. Kinetic analysis further revealed a b‐value of 0.97 (calculated from Equation (2)), higher than that of Ti_3_C_2_T_x_/SNFs (Figure 8j). The elevated *b*‐value can be directly linked to the improved electronic properties imparted by NCDs, which facilitate rapid charge transfer and minimize ion diffusion resistance within the MXene matrix.

In summary, CDs have emerged as a versatile modifier for pseudocapacitive materials, enhancing performance through tailored nanostructures. Their conductive sp^2^‐carbon cores (e.g., in GQDs) improve electron transport in metal oxides, while polar functional groups stabilize polymer electrodes. Heteroatom doping (N, S) introduces redox‐active sites, boosting areal capacitance and accelerating reaction kinetics. CDs also mitigate structural degradation in 2D materials and enable high‐energy‐density designs. By tuning crystallinity, doping, and surface chemistry, CDs bridge the gap between conductivity, stability, and redox activity in hybrid pseudocapacitors.

#### 4.1.3 CDs in Faradaic Capacitors

Currently, available battery‐type materials are primarily based on oxides, phosphides, sulfides, and hydroxides of Ni, Co, Cd, etc.^[^ ^135^, ^136^, ^137^, ^138^, ^139^, ^140^, ^141^, ^142^, ^143^, ^144^, ^145^, ^146^ ^]^ These materials often exhibit high storage capacity due to their multi‐valence redox abilities, drawing significant attention. However, in practical applications, these materials face dual bottlenecks: insufficient electrical conductivity and structural instability during cycling, which severely restrict their performance improvement and commercialization process.^[^ ^147^, ^148^ ^]^ CDs offer novel pathways to overcome these challenges through the construction of conductive networks, surface functionalization modification, and electronic structure regulation. Crystalline CDs prepared via top‐down methods (such as graphite‐derived CDs) can significantly enhance the electron transport efficiency in metal oxide/hydroxide systems. Heteroatom doping with N, S, etc., can introduce redox‐active sites and improve the wettability. Moreover, the atomic defects on the surface of CDs can form non‐covalent interactions with host materials, effectively maintaining the structural stability of the system.

CDs primarily serve as conductive bridges in metal oxide systems, with performance strongly dependent on their crystallinity and doping. Luo et al. used graphite oxide as raw material to synthesize CDs by hydrothermal treatment and then successfully prepared tremella‐like NiCo_2_O_4_/CDs composites by magnetic stirring.^[^ ^149^ ^]^ The resulting NiCo_2_O_4_/CDs composite exhibited exceptional electrochemical performance, including a high specific capacitance of 1242 F g^−1^ (compared to 790 F g^−1^ for pristine NiCo_2_O_4_) and an energy density of 38 Wh kg^−1^ at 800 W kg^−1^. The composite demonstrated outstanding cycling stability, retaining 99% of its initial capacitance after 4000 cycles, showcasing CDs' ability to stabilize the electrode structure during repeated redox reactions. In a contrasting approach highlighting the tunability of CDs, Wang et al. synthesized CDs with abundant hydrophilic groups via microwave pyrolysis using citric acid and ethylenediamine as precursors.^[^ ^101^ ^]^ This approach not only enhanced conductivity by 30‐fold but also improved wettability through surface oxygen‐containing groups, achieving a remarkable specific capacitance of 2202 F g^−1^. When configured into a symmetrical NiCo_2_O_4_/CDs EC, the device delivered impressive energy storage characteristics, achieving 73.5 Wh kg^−1^ at 499.98 W kg^−1^ and maintaining 62.5 Wh kg^−1^ even at a high power density of 5.0 kW kg^−1^. These results not only surpass the performance of conventional faradaic capacitors (typically 50–120 Wh kg^−1^ and 0.1‐10 kW kg^−1^) but also illustrate how CDs surface engineering can be strategically leveraged to optimize both energy and power density in advanced EC systems.

Transition metal hydroxides like α‐Ni(OH)_2_ (theoretical capacitance: 2365 F g^−1^) face intrinsic limitations of poor conductivity and structural instability that CDs effectively address through tailored modification strategies.^[^ ^150^ ^]^ Xia et al. revealed through density of states (DOS) analysis that the adsorption of CDs on Co(OH)_2_ induced significant electronic structure modifications.^[^ ^151^ ^]^ Bader charge analysis further indicated negligible charge transfer at the Co(OH)_2_/CDs interface, confirming non‐covalent interactions that preserve the high mobility of CDs. This mechanism enhances the electrical conductivity of CDs/Co(OH)_2_ electrodes by constructing conductive pathways without compromising the electrochemical activity of Co(OH)_2_. Sun et al. employed crystalline CDs obtained from graphite rods to boost the conductivity of α‐Ni(OH)_2_, developing CDs modified α‐Ni(OH)_2_ (α‐Ni(OH)_2_/CDs) using a one‐step hydrothermal method, which featured improved conductivity and an increased specific surface area.^[^ ^152^ ^]^ The optimal α‐Ni(OH)_2_/CDs electrode achieved a higher specific capacitance of 1724.0 F g^−1^ at 3 A g^−1^, 2.3 times higher than the bare α‐Ni(OH)_2_ electrode (750.7 F g^−1^). The morphology of hydroxides can be effectively regulated by introducing functional CDs during the electrodeposition process. Han et al. fabricated α‐Ni(OH)_2_/CDs thin film electrodes using cathodic electrodeposition.^[^ ^153^ ^]^ By incorporating functional CDs into the electrodeposition process, they enabled in situ morphological control of the hydroxide. In contrast to the relatively dense aggregates of undoped thin film electrodes, the resulting α‐Ni(OH)_2_/CDs material exhibited a porous structure, leading to enhanced electrochemical activity (155.57 mAh g^−1^ at 40 A g^−1^), with a capacity retention rate of 45.3%, compared to 24.3% for pure Ni(OH)_2_.

**Figure 9:**
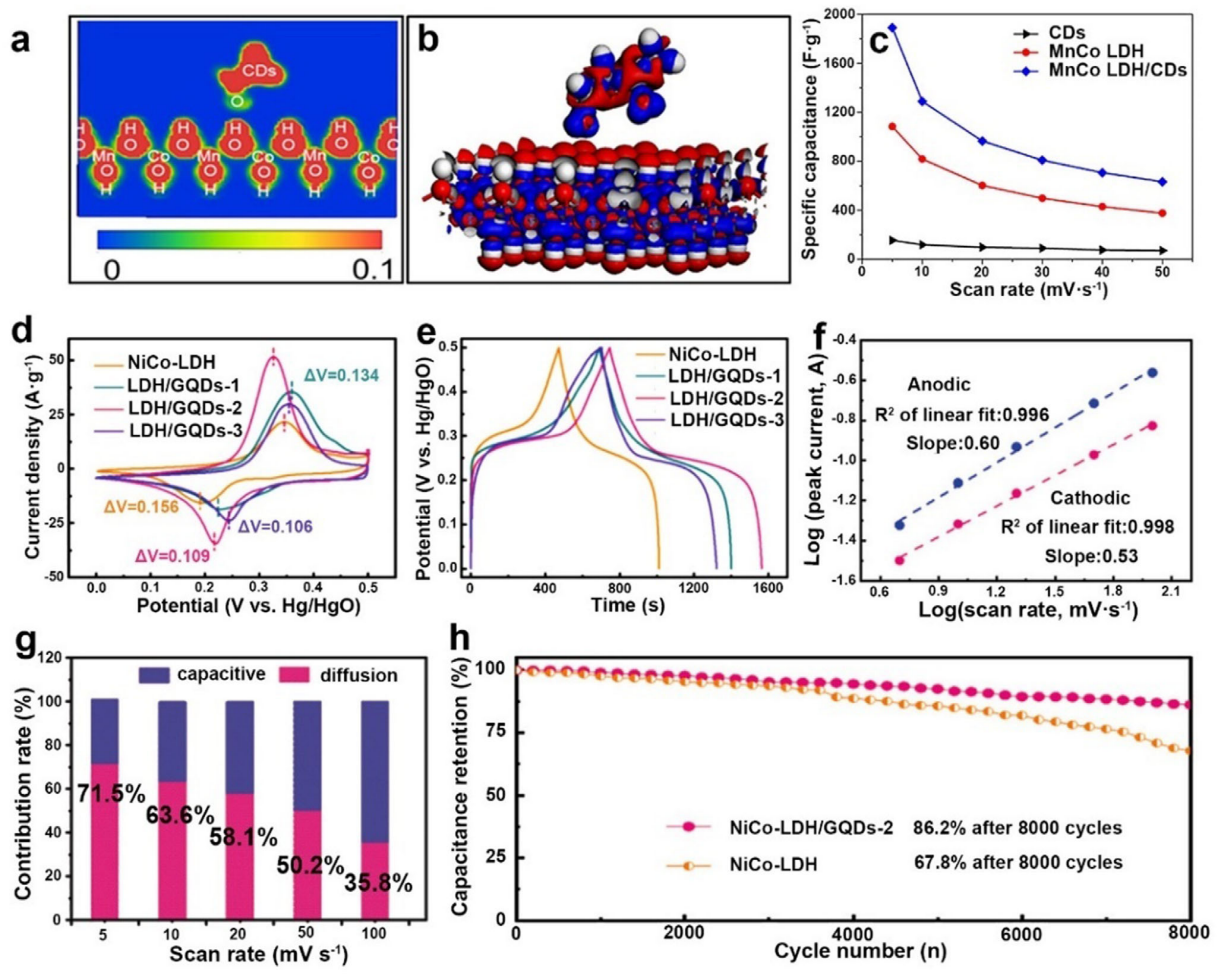
Using CDs as an additive to battery‐type electrodes. a) 2D slice view of electron localization function, b) spin‐charge density, and c) specific capacitance at different MnCo LDH/CDs scan rates. Reproduced with permission.^[^ ^155^ ^]^ Copyright 2021, American Chemical Society. d) CV curves, e) GCD curves at 1 A g^−1^, f) power‐law dependence of anodic and cathodic peak current and g) capacitive and diffusion‐controlled contribution at different scan rates, h) capacitance retention after 8000 cycles of the CDs@NiCo‐LDH. Reproduced with permission.^[^ ^66^ ^]^ Copyright 2022, WILEY‐VCH.

Layered double hydroxides (LDH), 2D structural materials with the general composition [M_1‐x_^2^ + M_x_^3+^(OH)_2_] [A^n−^]_x/n_ mH_2_O, are promising EC electrodes due to tunable composition and high redox activity.^[^ ^154^ ^]^ However, their layered structure tends to aggregate, hindering ion migration, while poor electrical conductivity limits performance. CDs address these issues by improving conductivity and reinforcing structural stability. Song et al. showed that CDs induce electronic structure reorganization in MnCo‐LDH, introducing new states near the Fermi level to enhance conductivity.^[^ ^155^ ^]^ The electron localization function (ELF) analysis demonstrated partially delocalized electrons on the ─OH groups of CDs (**Figure** **9**a,b), which served as charge transport mediators, facilitating efficient interfacial charge redistribution. This charge redistribution created a synergistic space charge compensation (SSC) effect, optimizing both charge transport kinetics and structural stability. The improved electronic coupling, combined with expanded ion diffusion channels, led to exceptional electrochemical performance, resulting in a specific capacitance of approximately 1890 F g^−1^ for the composite (Figure 9c).

While Song et al. focused on enhancing conductivity through interfacial electron states near the Fermi level, Zhao et al. leveraged the 2D conductive network of GQDs to establish a well‐connected electric field network on the surface of LDH.^[^ ^66^ ^]^ The integration of GQDs with NiCo‐LDH (NiCo‐LDH/GQDs) induced significant electronic structure modifications, enhancing charge transfer dynamics through interfacial electron donation from the sp^2^‐carbon network of GQDs to Ni/Co metal centers. This process established an electron‐rich environment at the heterointerfaces, generating a robust built‐in electric field that elevated the composite density of states at the Fermi level while reducing the charge transfer bandgap. These synergistic effects lowered the OH^−^ adsorption energy by 0.37 eV, collectively accelerating interfacial charge transfer kinetics and enabling a specific capacitance of 1628 F g^−1^. Electrochemical characterization revealed distinct faradaic behavior in NiCo‐LDH/GQDs, with CV curves displaying clear redox peaks (Figure 9d) and GCD curves showing two plateaus (Figure 9e). The decreasing potential difference between redox peaks in CV curves with GQDs addition indicated improved reaction kinetics and electrochemical reversibility. B‐value calculations (0.60 for the anode and 0.53 for the cathode, Figure 9f) confirmed coexisting capacitive and diffusion‐controlled processes (Figure 9g). Notably, NiCo‐LDH/GQDs retained 86.2% of its capacitance after 8000 cycles at 50 mV s^−1^, far exceeding the stability of bare NiCo‐LDH (Figure 9h).

Transition metal sulfides (TMS) such as CuS,^[^ ^156^ ^]^ CoS_2_,^[^ ^157^ ^]^ Ni_3_S_2_,^[^ ^158^ ^]^ NiCo_2_S_4_^[^ ^159^ ^]^ exhibit higher electrical conductivity and electrochemical activity than many metal oxides/hydroxides, but their practical capacitor applications are limited by volume expansion‐induced cycling instability and poor rate performance.^[^ ^62^, ^160^ ^]^ CDs have been engineered into TMS composites to address these challenges. Arsalani et al. synthesized CDs‐modified CoS_2_ nanocomposites via ball‐milling‐assisted hydrothermal methods, yielding mesoporous structures with high specific surface areas that outperformed pure CoS_2_ in capacitance, charge transport, and cycling stability.^[^ ^157^ ^]^ Jia et al. electrodeposited NiCo_2_S_4_/N‐GQDs composites, where N‐GQDs synergized with the unique honeycomb structure of NiCo_2_S_4_ to improve capacitance performance and structural stability.^[^ ^161^ ^]^ The tiny N‐GQDs served as conductive bridges between adjacent NiCo_2_S_4_ nanosheets, establishing novel electron transport pathways. The assembled symmetric ECs exhibited a high energy density of 127 µWh cm^−1^ at a power density of 1000 µW cm^−2^, demonstrating the synergistic advantage of N‐GQDs in optimizing both electrochemical kinetics and structural integrity for faradaic energy storage systems. Lu et al. developed Ni‐Co‐Se/NCDs hollow microspheres via a simple hydrothermal synthesis, in which N‐CDs derived from KB served as conductive agents to accelerate electrochemical kinetics, regulate particle size, and shorten ion transport paths.^[^ ^93^ ^]^ The synthesis involved a core‐shell growth mechanism where N‐CDs chelated with Ni/Co ions and anchored onto Se cores, forming a hollow architecture that synergized high conductivity with fast ion diffusion. This composite achieved a specific capacity of 151.1 mAh g^−1^ at 1 A g^−1^ and 84.7 mAh g^−1^ at 100 A g^−1^. The resulting HSC achieved a maximum energy density of 41.1 Wh kg^−1^ at 191.5 W kg^−1^ and a power density of 38.3 kW kg^−1^ at 23.1 Wh kg^−1^. These studies highlight CDs as versatile modifiers for enhancing TMS‐based energy storage materials through conductivity improvement, structural stabilization, and kinetic optimization.

In addition to being used for modifying faradaic materials, CDs have also been reported to exhibit pseudocapacitive behavior themselves. Recent research has shown that some carbon‐based nanomaterials can exhibit unusual pseudocapacitive and faradaic behavior through modification and functionalization. Pallavolu et al. synthesized N‐doped and S‐doped CDs using citric acid as the carbon source, adding melamine and thiourea as the N and S sources, respectively.^[^ ^74^ ^]^ The abundant functional groups containing O‐/N‐/S in these CDs provided redox‐active sites, exhibiting unusual faradaic behavior (strong oxidation‐reduction peaks in CV curves). Pure CDs, N‐CDs, and S‐CDs materials achieved specific capacities of 125, 181, and 284 mAh g^−1^ at 1 A g^−1^. Symmetric ECs based on S‐CDs electrodes obtained a specific capacitance of 21.7 mAh g^−1^, an energy density of 15.2 Wh kg^−1^, and a power density of 700 W kg^−1^.

CDs enhance faradaic capacitors by integrating structural and chemical functionalities: crystalline cores for conductivity, heteroatoms for redox activity, and defects/groups for structural stability. This multi‐mechanistic approach addresses the core limitations of metal compounds, offering a pathway to high‐energy‐density devices with prolonged cycle life.

### 4.2 CDs as Electrolyte Additives

In ECs, electrolytes are crucial for power density, rate capacity, internal resistance, and cycle stability, since electrolytes regulate ionic conductivity and determine the potential window of ECs.^[^ ^6^ ^]^ Moreover, the interaction between electrodes and electrolytes significantly influences the charge storage process. CDs are promising additive materials for ECs due to their cost‐effectiveness, well‐dispersion, and environmentally friendly compared to other chemical additives.^[^ ^162^, ^163^ ^]^

Electrolytes in ECs can be classified into several categories: aqueous‐based, organic, ionic liquids, solid or quasi‐solid electrolytes, and mixed electrolytes. Among these, aqueous‐based electrolytes, such as KOH, H_2_SO_4_, and Na_2_SO_4_, are popular due to their low cost, nonflammability, and high ionic conductivity. However, they are plagued by water splitting, which limits the working voltage and energy density. The surface functional groups of CDs have a strong affinity for cations in the electrolyte, improving the capacity and energy density of the ECs with aqueous electrolytes. Hou et al. reported that introducing egg yolk‐derived CDs in 6 M KOH electrolyte expanded the voltage window from 1 to 1.8 V, resulting in a specific energy of about 51.4 Wh kg^−1^ and a specific power of ≈450 W kg^−1^.^[^ ^164^ ^]^

**Figure 10:**
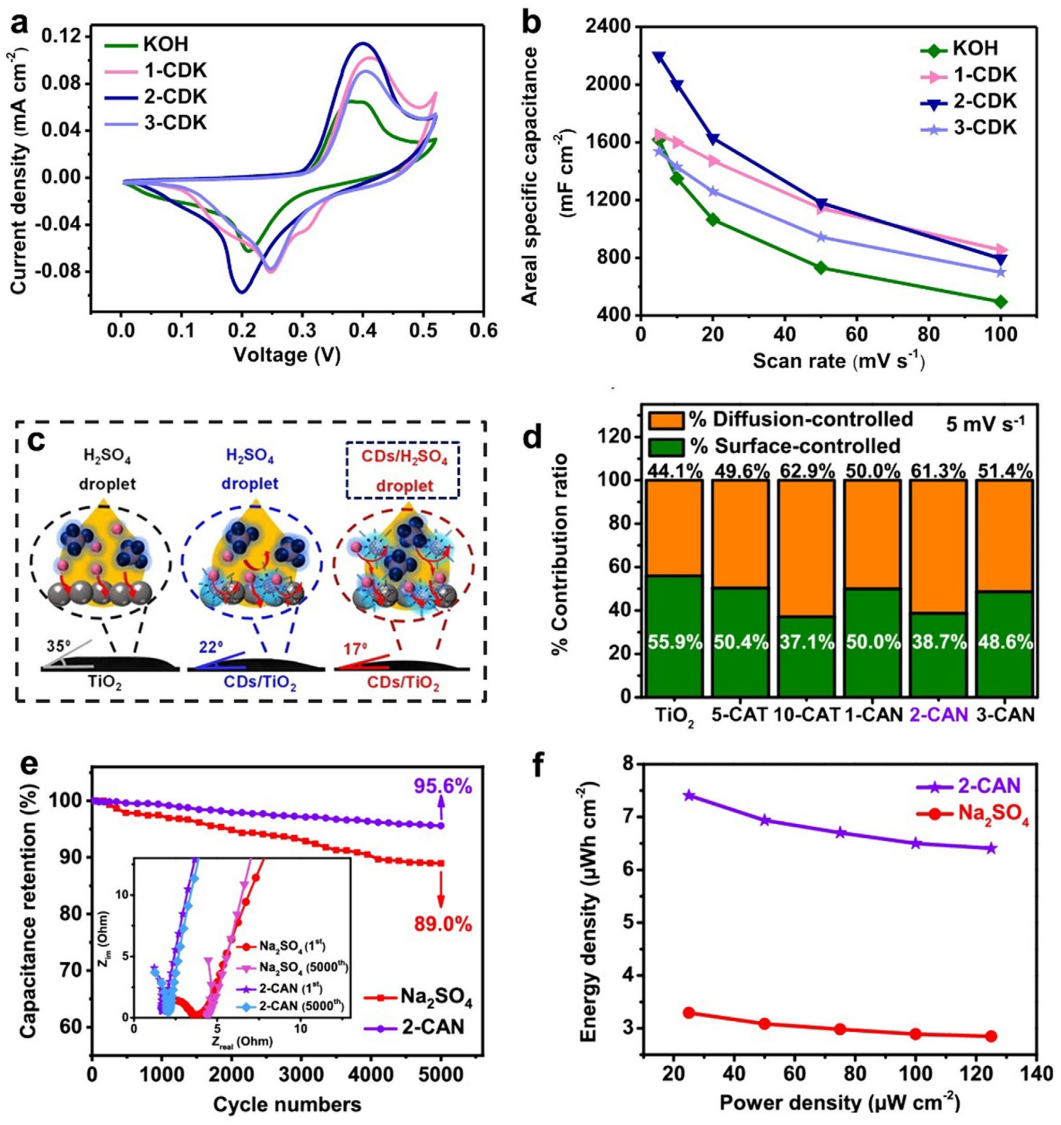
Using CDs as an additive to aqueous electrolyte. a) CV curves and b) a plot between specific capacitance and scan rate of TiO_2_/CDs of the electrode in KOH and KOH/CDs solution. Reproduced with permission.^[^ ^29^ ^]^ Copyright 2021, Elsevier. c) Contact angles and schematic representation of electrodes using H_2_SO_4_ solution with and without CDs. Reproduced with permission.^[^ ^165^ ^]^ Copyright 2022, Elsevier. d) Charge transfer contribution, e) Capacitance retention and EIS curves change (inset), f) Ragone plot of electrodes system in Na_2_SO_4_ and Na_2_SO_4_/CDs solution. Reproduced with permission.^[^ ^166^ ^]^ Copyright 2022, American Chemical Society.

Paoprasert and co‐workers discovered that incorporating CDs rich in oxygen‐containing functional groups derived from sodium polyacrylate into KOH, H_2_SO_4_, and Na_2_SO_4_ electrolytes significantly enhanced ion dissociation, kinetics, and overall electrochemical performance of electrodes. The abundant oxygen‐containing groups (e.g., carboxyl, hydroxyl) on CDs’ surfaces improve electrolyte wettability at the electrode/electrolyte interface, enabling faster faradaic reactions. For instance, adding CDs to KOH (CDK) boosted the specific capacitance of TiO_2_/CDs electrode by 133% compared to pure KOH (**Figure** **10**a,b).^[^ ^29^ ^]^ CDs enhanced the wettability and hydrophilicity at the electrode/electrolyte interface, improving the capacitance performance of ECs (Figure 10c).^[^ ^165^ ^]^ Pholauyphon et al. further showed that pyrolyzed sodium polyacrylate CDs added to a neutral Na_2_SO_4_ electrolyte increased capacitance by 229%, with the diffusion contribution to charge storage rising from 54.4% to 71.8% (Figure 10d).^[^ ^166^ ^]^ Using the CDs/Na_2_SO_4_ system as an electrolyte decreased the solution resistance, significantly improving cycle stability (96% retention after 5000 cycles, Figure 10e). ECs with TiO_2_/CDs electrodes in the CDs/Na_2_SO_4_ electrolyte achieved higher energy and power density, measuring 7.4 µWh cm^−2^ and 25 µW cm^−2^, respectively (Figure 10f).

**Figure 11:**
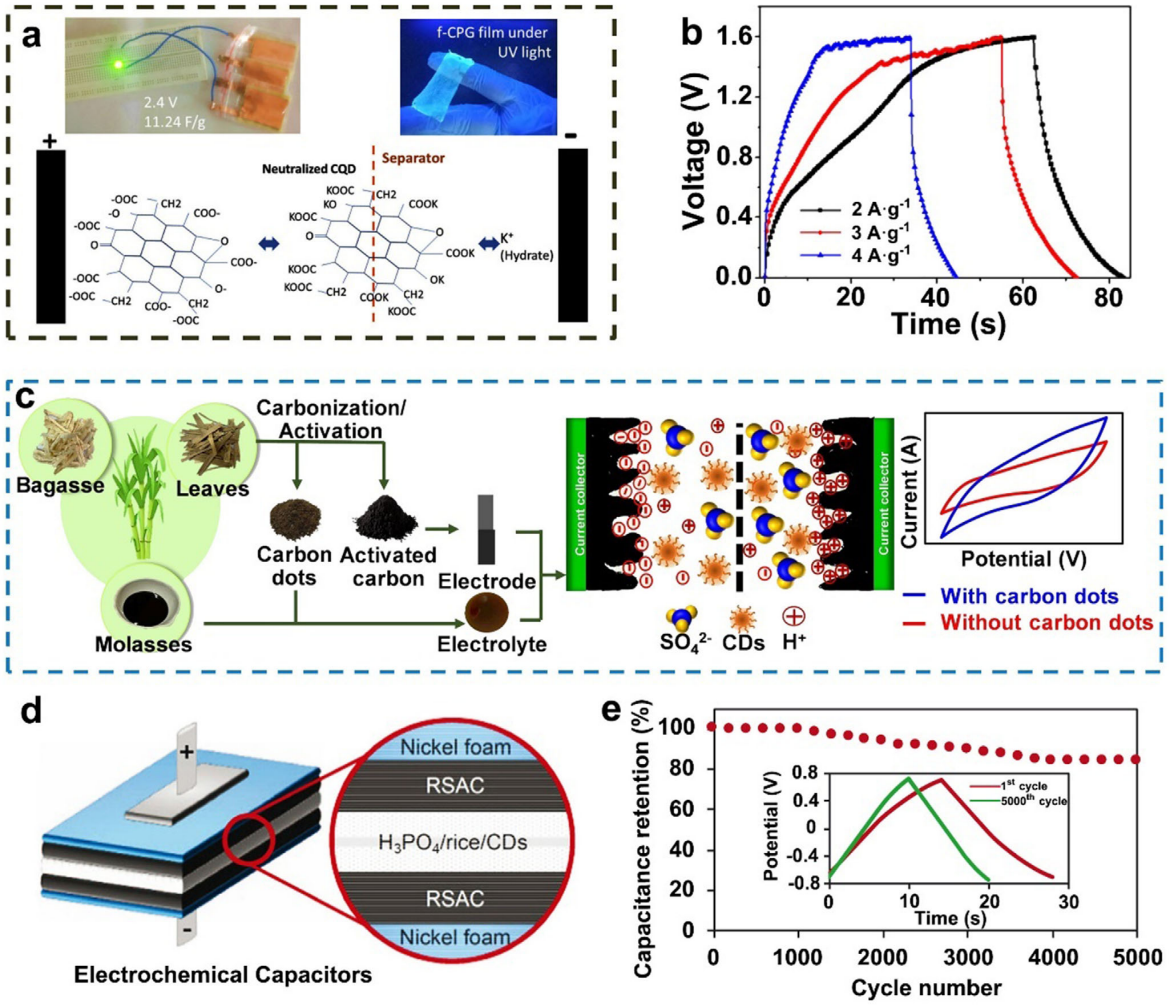
Using CDs as an additive to solid, quasi‐solid, or mixed electrolytes. a) Photos of CD‐based polymer gel, the fabricated device, and b) their GCD profiles. Reproduced with permission.^[^ ^167^ ^]^ Copyright 2022, Elsevier. c) The schematic representation of the preparation of bio‐renewable CDs and the ECs systems with and without CDs. Reproduced with permission.^[^ ^168^ ^]^ Copyright 2022, WILEY‐VCH. d) Schematic representation of the CD‐based quasi‐solid ECs, and e) their capacitance retention curves and GCD curves before and after 5000 cycles. Reproduced with permission.^[^ ^169^ ^]^ Copyright 2021, Elsevier.

CDs alone can also be used as an electrolyte. The ionic conductivity of a 25 mM CD solution was measured to be 0.43 S cm^−1^, which was higher than that of a 1 M Na_2_SO_4_ solution.^[^ ^167^ ^]^ When using pure CD solution as the electrolyte, the graphene nanosheet electrodes achieved a weight capacitance of 155 F g^−1^ at 1 A g^−1^. Kumar et al. added carboxymethyl cellulose to the CD suspension to create a CD‐based polymer gel film. They then used this film to prepare a solid‐state capacitor with graphene electrodes, where the CD polymer gel film acted as both the electrolyte and diaphragm (**Figure** **11**a). The solid‐state ECs exhibited low leakage current and maintained a stable open circuit potential (self‐discharge), a specific capacitance of 140 F g^−1^ at 2 A g^−1^, and a 1.6 V working voltage (Figure 11b).

CDs with abundant surface functional groups, derived from biomass materials, are readily dispersed in organic solutions and can be utilized in organic electrolytes to enhance their electrical conductivity. Paoprasert et al. attempted to use a renewable electrolyte (organic/water mixed electrolyte) for ECs. They fabricated CDs from rice husk, cassava peel, and sugarcane bagasse as electrolyte additives to enhance the electrolyte's conductivity and the electrode's wettability. The obtained CDs were used in molasses‐H_2_SO_4_,^[^ ^168^ ^]^ cassava starch‐H_2_SO_4_,^[^ ^34^ ^]^ and rice starch‐H_3_PO_4_^[^ ^169^ ^]^ electrolytes to achieve specific capacitance of approximately 491, 375, and 456 F g^−1^, respectively.

Thumkaew et al. prepared CDs from bagasse and leaves. CDs' heteroatoms and surface functional groups serve as active centers, facilitating ion dissociation and ion transport, thereby improving the capacitive performance of the molasses electrolyte system (Figure 11c).^[^ ^168^ ^]^ The EIS test indicated that after introducing CDs, the curves in the low‐frequency region were more vertical. The plot shifted to the smaller real impedance, showing that adding CDs improved the surface capacitance contribution and reduced the resistance and leakage current. Jorn‐am et al. prepared the quasi‐solid‐state ECs with rice starch‐H_3_PO_4_ quasi‐solid electrolyte and CDs additive (Figure 11d).^[^ ^169^ ^]^ The capacitance retention rate of ECs was 84% after 5000 cycles, and the electrolyte still maintained the quasi‐solid‐state morphology after 8 weeks (Figure 11e). While research on CDs as electrolyte additives in ECs is limited, the results above indicate that CDs as electrolyte additives can enhance capacitor performance and extend service lives.

In summary, CDs demonstrate exceptional versatility as next‐generation nanomaterials across diverse energy storage applications, including their roles in electrode materials for EDLCs, pseudocapacitors, battery‐type electrodes, and electrolyte additives. Their unique attributes—structural tunability, surface chemistry richness, and distinct electronic properties—synergistically enhance key performance metrics such as capacitance, cycle stability, and energy density, addressing critical challenges in conventional energy storage systems.

Unlike traditional carbon nanomaterials, CDs integrate multifaceted enhancement mechanisms across all components of energy storage devices: forming conductive networks in electrodes, stabilizing structures via non‐covalent interactions, activating redox sites through surface chemistry, and mediating ion solvation in electrolytes. This integrative capability positions CDs as a foundational element for developing high‐performance, sustainable electrochemical systems, ranging from high‐power EDLCs to long‐lasting battery‐type devices.

To contextualize these advancements, **Table** **1** systematically compares the performance of CDs‐enhanced ECs, summarizing key characteristics including carbon precursors, synthesis methods, specific capacitance, cycling stability, power density, and energy density of various CDs‐based composite materials.

**Table 1:** Performance of ECs based on CDs as electrode material and electrolyte additive.

Composite Materials	Synthesis of CDs		Electrolyte	Capacitance (F g^−1^ at 1 A g^−1^)		Capacity retention (10 000 cycles)		Power density (W kg^−1^)	Energy density (Wh kg^−1^)	Refs.
	Precursor	Method		Initial	With CDs	Initial	With CDs			
AC/CDs	Cassava peel	Hydrothermal	—	138	239.5	66.4%	85.7%	—	—	[34]
AC/CDs	Citric acid and urea	Solid‐phase microwave	2 m H2SO4	125.8	301.7	85%	99.5%	63	42	[100]
CDs embedded AC	Bituminous coal powder	Chemical oxidation	6 m KOH	≈250	354	—	≈100%	125	13.47	[114]
Edge‐CNFs	Coal powder	Chemical oxidation	6 m KOH	≈150	200	—	98%	127.5 mW cm^−2^	70.83 µWh cm^−2^	[98]
CDs/CNFs	Graphene oxide sheet	Photo‐Fenton reaction	1 m H2SO4	252	319	—	—	26	6.4	[115]
GO/BLs/CDs	Chitosan	Hydrothermal	1 m H2SO4	155	308	—	93.5%	150	13.4	[122]
GDs_2_/GO	Chitosan	Microwave hydrothermal	1 m H2SO4	176	324	—	96%	‐	‐	[121]
GDs/Gr	lignin	In situ activation	6 m KOH	162	404.6	—	97%	225	35.1	[47]
CPD@HCPOne(CC)	Cyanuric chloride	Hydrothermal	1 m TEATFB/PC		150.4	—	99.78%	37 497	35.8	[28]
CDs/CC	Citric acid and urea	Hydrothermal	PVA/ H2SO4 gel	—	844	—	71.9%	200 mW cm^−2^	17.04 µWh cm^−2^	[99]
CDs‐matrix structures	Tofu‐dreg wastes	Pyrolysis	1 m H2SO4	—	500	—	98%	124	10.7	[35]
CDs@MnO_2_	Graphene oxide	Hydrothermal	EMIMBF4	148.4	208.2	56.1%	82.4%	11 600	82.2	[21]
RuO_2_‐Trp‐CDs‐GO	Citric acid and tryptophan	Hydrothermal	0.5 m Na2SO4	—	503.7	—	99.5%	52 000	332	[53]
N‐CDs@PANI	Citric acid	Microwave	1 m H2SO4	274	785	60% (1200)	70% (2000)	400	49.9	[170]
PANI/CDs	PEG	Hydrothermal	PVA/ H2SO4 gel	220	380	50% (1000)	77% (3000)	197	52	[171]
	Urea and glycine	Hydrothermal		220	870	50% (1000)	84.5%(1000)	200	121	
MXene/CDs	Calcium alginate gel	Pyrolysis	3 m H2SO4	319.0	372.6	—	95.0%	13.1 kW L^−1^	27.2 Wh L^−1^	[57]
CDs@Ti_3_C_2_T_x_	Chitosan	Hydrothermal	1 m H2SO4	277	441.3	—	96.2%	350	25.8	[172]
NiCo_2_O_4_@CDs	GO power	Hydrothermal	2 m KOH	790	1242	—	99%	800	38	[149]
CDs/NiCo_2_O_4_	Citric acid and ethylenediamine	Microwave thermolysis	1 m Na2SO4	≈400	2202	—	95.37%	499.98	73.5	[101]
CDs/α‐Ni(OH)_2_	Graphite rods	Electrochemical etching	3 m KOH	750.7 (3 A g^−1^)	1724.0 (3 A g^−1^)	—	99.91%	3000	44	[152]
Ni(OH)_2_‐CDs	Acetaldehyde	Hydrothermal	1 m KOH	1710.2 (5 A g^−1^)	2030.2 (5 A g^−1^)	24.3% (1000)	45.3% (1000)	45 000	77.5	[153]
CDs‐NiCo‐LDH	Commercial CDs	—	2 m KOH	1769	2220	<60% (5000)	79.2% (5000)	8000	50.84	[173]
CDs‐MnCo‐LDH	Ethanol	Electrochemical anodizing	1 m LiOH	≈1100	2063	30.3% (3000)	74.1% (3000)	666	79	[155]
CDs/CoS_2_	Glycerol	Microwave	3 m KOH	448	808	—	98.75%	‐	‐	[157]
CuS@CDs	Citric acid and ethylenediamine	Hydrothermal	6 m KOH	434.5 (0.5 A g^−1^)	920.5 (0.5 A g^−1^)	—	92.8%	397.75	44.19	[156]
CDs/Ni_3_S_2_	Carbon nanotubes	Chemical oxidation	6 m KOH	980 (2 A g^−1^)	1130 (2 A g^−1^)	—	80%	134	18.8	[158]
NCDs/HCNT/MoS_2_	Citric acid and thiourea	Hydrothermal	1 m Na2SO4	≈2750 mF cm^−2^	3360 mF cm^−2^	—	89.2% (2500)	5687 µW cm^−2^	673 µWh cm^−2^	[132]
N,S‐CDs/rGO/NiCo_2_S_4_	Graphite powder	Chemical oxidation	2 m KOH	≈145	162.6	81.7%	87.5%	14 400	51.0	[159]
NCDs‐NiCo_2_S_4_	Pyrene and nitric acid	Hydrothermal	1 m PVA‐KOH	1100 mC cm^−2^	1803 mC cm^−2^	—	83% (2500)	1000 µW cm^−2^	127 µWh cm^−2^	[161]
TiO_2_/CDs	Sodium polyacrylate	Pyrolysis	1 m Na2SO4 (with /without CDs)	108 mF cm^−2^	247 mF cm^−2^	89.0% (5000)	95.6% (5000)	25 µW cm^−2^	7.4µWh cm^−2^	[166]
TiO_2_/CDs	Sodium polyacrylate	Pyrolysis	2 m KOH (with/ without CDs)	1661 mF cm^−2^	2200 mF cm^−2^	86.95% (5000)	91.43% (5000)	635 µW cm^−2^	51.3 µWh cm^−2^	[29]
TiO_2_/CDs	Sodium polyacrylate	Pyrolysis	2 m H2SO4 (with/ without CDs)	121 mF cm^−2^	643 mF cm^−2^	90.9% (5000)	98.4% (5000)	187.5 µW cm^−2^	24.3 µWh cm^−2^	[165]

Notably, despite these promising advancements, the performance inconsistencies occurred across current studies, such as divergent capacitance values (e.g., 301.7–388 F g^−1^ in EDLCs, 133–229% enhancement in electrolytes), indicating critical methodological and material‐related limitations. These discrepancies stem from three interconnected factors: 1) the structural difference of fabricated CDs. Due to the synthesis routes (top‐down vs bottom‐up) and reaction precursors (crystalline carbon, carbon‐containing molecules, biomass etc.), the structures of fabricated CDs vary in surface functional groups, crystallinity, and doping, significantly affecting CD's interaction with electrodes/electrolytes; 2) divergent fabrication protocols of electrodes or electrolytes, including CDs loading ratios, dispersion methods (in situ growth vs physical mixing), and electrode processing conditions, resulting in the performance variability of capacitors; 3) inconsistent reporting standards, such as the use of gravimetric versus volumetric capacitance or variable cycling parameters (current density, cycle number). These factors contribute to divergent or even conflicting results when evaluating the performance of capacitors in various reports.

Addressing these inconsistencies requires explicit characterization of CDs’ key properties (e.g., surface group density via XPS, crystallinity via Raman), detailed documentation of synthesis parameters for reproducibility, and adoption of unified electrochemical testing protocols. Using systematic and comparative experimental approaches, researchers can resolve conflicting results, uncover generalizable design principles, and fully harness CDs’ potential to bridge performance gaps in next‐generation energy storage systems.

## 5 CDs for Photo‐Assisted ECs

Integrating light energy with electrochemical systems has emerged as a transformative research avenue in the quest to augment EC's energy storage capabilities.^[^ ^174^, ^175^, ^176^, ^177^ ^]^ Early efforts to develop photorechargeable storage devices primarily centered on two strategies: externally coupling light‐harvesting components, such as Si solar cells or perovskite solar cells, with storage units like Li‐ion capacitors, batteries, or carbon‐based ECs; and fabricating solar cells and energy storage devices in a layer‐by‐layer configuration on a common electrode. While these designs achieved compactness and reduced weight, they were plagued by phase mismatches, energy leakage, and complex architectures.

To address these challenges, photo‐assisted ECs have been developed, which leverage semiconducting materials to synergize solar energy harvesting and electrochemical storage through precise bandgap engineering and charge transfer dynamics. In recent years, research focus has shifted from traditional metal‐based semiconductors to unconventional nonmetallic materials, with CDs emerging as a promising candidate due to their unique combination of optical and electrochemical properties—as detailed in Section 3.3, CDs exhibit broadband light absorption (tuned by quantum confinement and surface defects) and multifunctional charge storage (integrating electric double‐layer capacitance and pseudocapacitance from surface redox reactions), making them ideal for seamless integration of light‐to‐energy conversion and electrochemical storage.

Photo‐assisted ECs represent an advanced class of energy storage systems that unify light energy conversion and electrochemical storage in a single device. CDs’ strong light absorption across a broad spectral range and large specific surface area facilitate efficient charge transfer. Upon photoexcitation, CDs generate electron‐hole pairs that serve dual functions: enhancing charge storage capacity by providing additional charge carriers and accelerating charge transfer kinetics at electrode–electrolyte interfaces. The synergistic combination of their fluorescent properties, conductivity, and photo‐responsiveness significantly improves light absorption efficiency and charge transfer rates, enabling direct solar‐to‐electrical energy storage—overcoming traditional ECs’ reliance on external charging sources.

### 5.1 CDs for Enhanced Charge Generation and Separation

As photoactive materials, CDs play crucial roles in photo‐assisted ECs by leveraging their intrinsic photoelectrochemical properties (Section 3.3). Their broadband absorption (UV to visible) arises from surface defect states (e.g., oxygen vacancies, N/S doping) and functional groups (─OH, ─COOH), which introduce localized energy levels in the bandgap and extend absorption to longer wavelengths, aligning with the solar spectrum.

Photoexcitation generates electron–hole pairs, with separation efficiency governed by surface chemistry and defects: defects trap electrons to suppress recombination (evidenced by lower PL intensity in defect‐rich CDs), while surface functional groups act as active sites for reversible redox reactions with electrolytes (e.g., H^+^, OH^−^), contributing pseudocapacitance. The dual mechanisms: defects boosting charge availability and functional groups enabling storage, synergistically enhances capacitance under illumination.

Some CDs also exhibit photothermal conversion capabilities, where localized heating under illumination reduces electrolyte viscosity and accelerates ion diffusion. In GQDs/MXene composites, heteroatom doping‐induced defects in GQDs not only suppressed MXene stacking but also amplified photothermal effects, locally heating the electrode to accelerate ion diffusion, thereby further contributing to capacitance enhancement alongside charge separation effects.^[^ ^30^ ^]^

**Figure 12:**
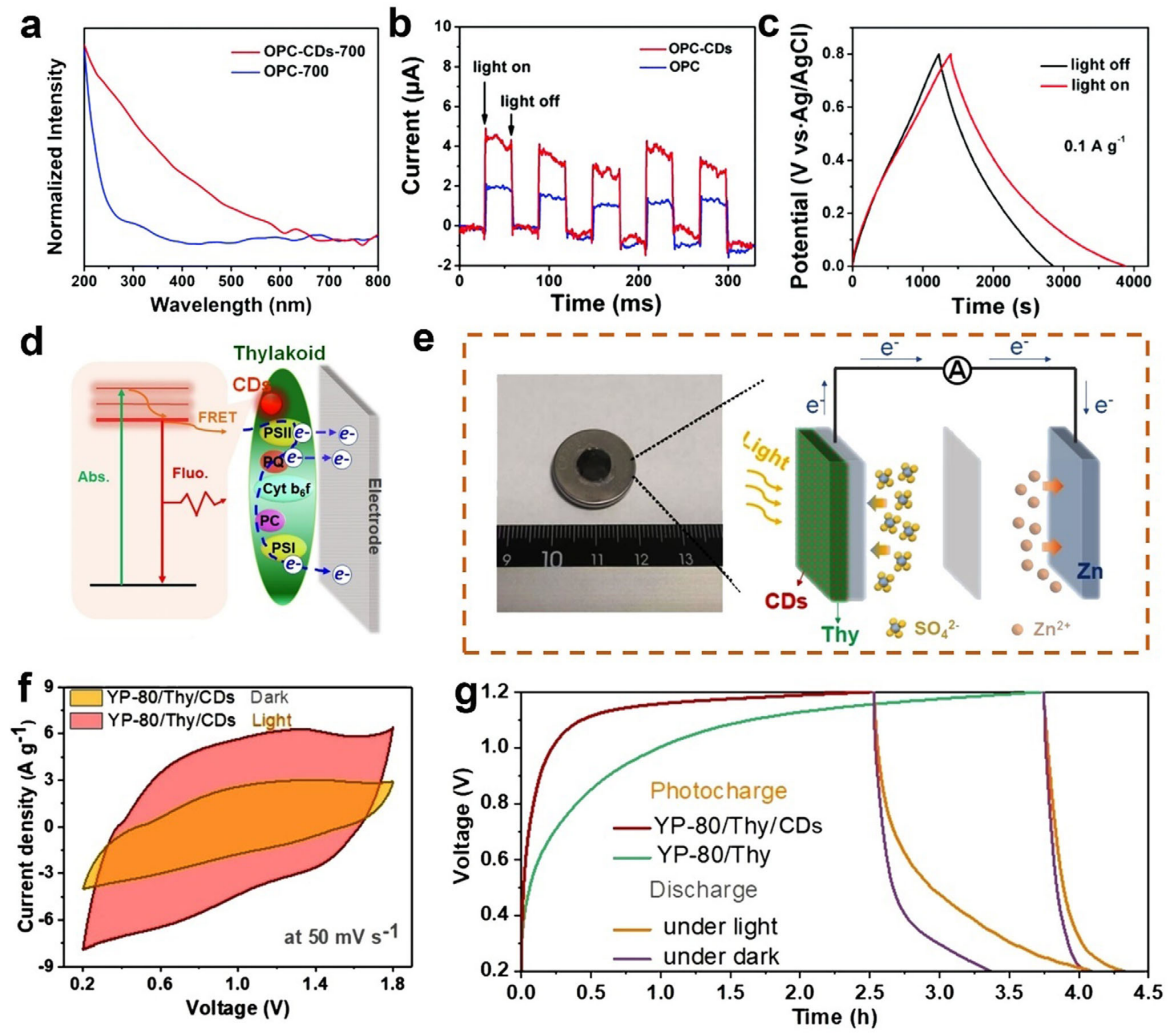
Using CDs as a photo‐carrier generation additive in photo‐assisted capacitors. a) UV–vis spectra, b) transient photocurrent responses, (c) GCD curves of OPC‐CDs materials. Reproduced with permission.^[^ ^28^ ^]^ Copyright 2020, Royal Society of Chemistry. d) Schematic illustration of the semi‐artificial photosynthesis process, e) Photograph (left) and schematic illustration (right), f) CV curves, and g) photo‐charge and galvanostatic discharge curves at 0.1 A g^−1^ of photo‐charging ZHCs using YP‐80/Thy/CDs cathode. Reproduced with permission.^[^ ^32^ ^]^ Copyright 2024, WILEY‐VCH.

When integrated with EC electrode materials, CDs can significantly enhance the photoresponsive behavior of the composite and ultimately improve device performance. The multifunctional nature of CDs as photo‐electrochemical materials is exemplified by several recent studies. Wang et al. developed photo‐assisted rechargeable ECs by combining CDs with procyanidins electrodes (OPC‐CDs).^[^ ^28^ ^]^ The achieved OPC‐CDs‐700 material had stronger visible light absorption (**Figure** **12**a) and higher photocurrent than the electrode without CDs (Figure 12b). After optimizing the concentration of CDs, the OPC‐CDs‐700 materials achieved a specific capacitance of 312 F g^−1^ at 0.1 A g^−1^ under illumination (Figure 12c), representing a substantial improvement over conventional EC materials. Sinha et al. demonstrated the effectiveness of CDs in UV‐activated systems by decorating zinc oxide and single‐walled carbon nanotube (SWCNT) composites with hydrothermally synthesized CDs.^[^ ^178^ ^]^ The resulting hybrid material showed a 41.38% enhancement in areal capacitance under UV illumination, as photogenerated electrons from the CDs participated actively in the photo‐assisted EC system.

Perhaps most remarkably, Qu et al. developed a novel photo‐rechargeable zinc‐ion hybrid capacitor (ZHC) by combining light‐absorbing CDs with natural thylakoid materials (CDs/Thy).^[^ ^32^ ^]^ This bio‐inspired system leveraged Förster resonance energy transfer (FRET) to boost photoelectron production, with the CDs/Thy hybrid converting absorbed green light into red light emission(Figure 12d). The photocurrent output of this hybrid photosystem was six times greater than that of the original thylakoid material, and when implemented as a photocathode in ZHCs under sunlight (Figure 12e), the CD‐modified system showed a 144% (Figure 12f) capacitance increase and achieved a photo‐charging voltage response of 1.2 V (Figure 12f).

To design device architectures, Sriramadasu et al. integrated red‐emitting carbon dots (RCDs) and Ni‐doped 2D MoS_2_ (NMS) into a PVA gel matrix to fabricate a symmetric photorechargeable EC.^[^ ^33^ ^]^ In this 0D/2D heterostructure, the RCDs functioned as efficient visible‐light harvesters and photosensitizers while the NMS provided robust capacitive performance, resulting in a system that achieved 13.35 mF cm^−2^ (light) and 5.61 mF cm^−2^ (dark), while maintaining excellent capacitance retention of 97.25%. **Table** **2** summarizes the performance of representative CDs‐based photo‐assisted ECs, demonstrating their enhanced capacitance under illumination compared to dark conditions. Key metrics include specific capacitance, light conditions, and improvement ratios, showcasing the versatility of CDs in diverse device configurations.

### 5.2 CDs for Photo‐Induced Charge Transfer and Storage

**Figure 13:**
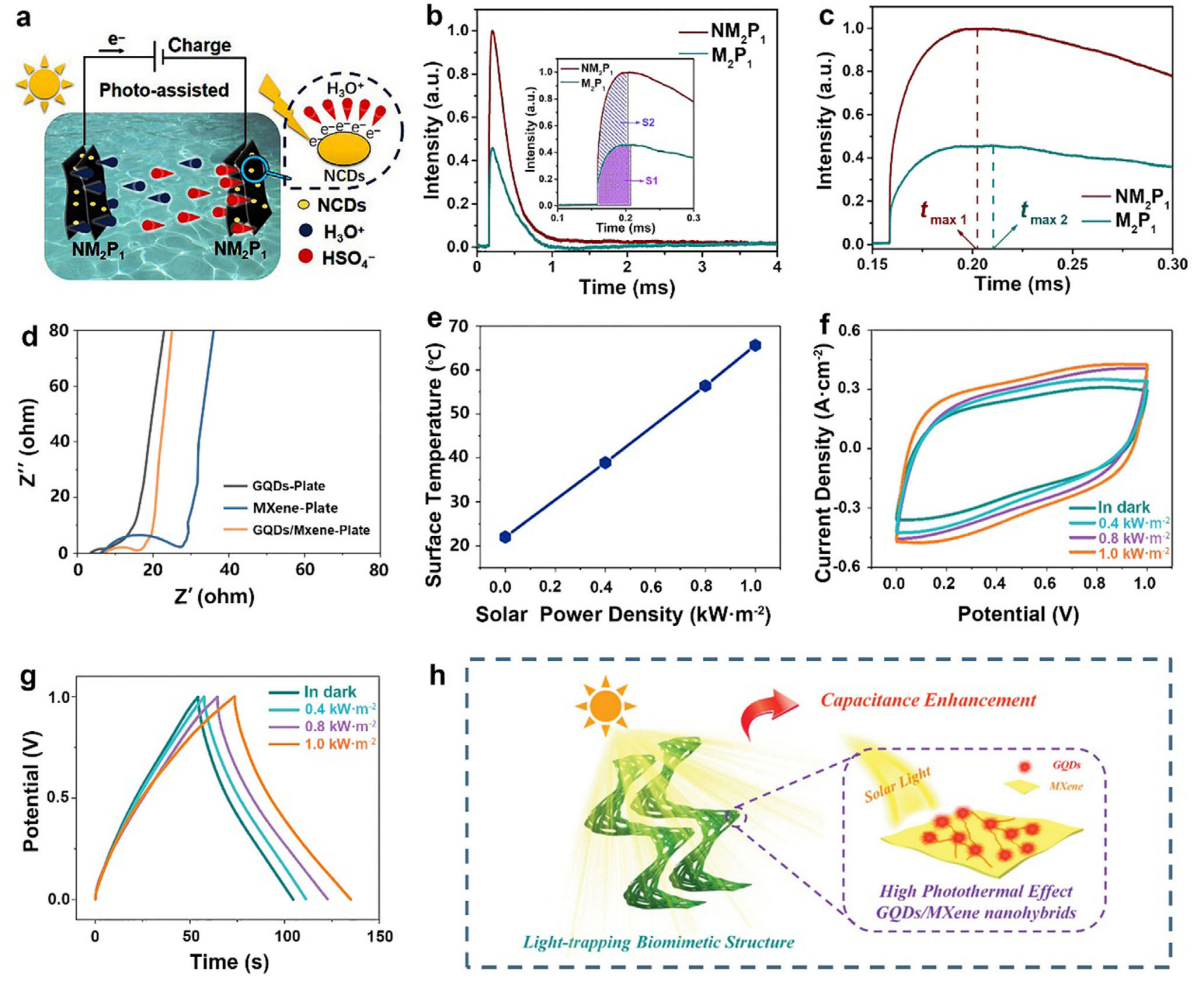
Using CDs as photo‐induced charge transfer and storage materials in photo‐assisted capacitors. a) A schematic diagram of the working mechanism, b) TPV patterns and the corresponding charge extraction process (inset), c) the enlarged view of maximum peak location (*t*_max_) of CDs/MXene fiber. Reproduced with permission.^[^ ^179^ ^]^ Copyright 2021, Springer Nature. d) EIS Nyquist plots, e) surface temperature, f) CV curves, g) GCD curves, and h) schematic diagram of enhancement of photothermal driving capacitance for CDs/MXene photothermal ECs. Reproduced with permission.^[^ ^30^ ^]^ Copyright 2022, WILEY‐VCH.

Beyond their role in charge generation and separation, CDs have demonstrated remarkable capabilities in photo‐induced charge transfer and storage processes that further enhance system performance. Wang et al. provided compelling evidence of this phenomenon through their work with NCDs modified fibrous Ti_3_C_2_T_x_ MXene materials.^[^ ^179^ ^]^ The resulting NCDs/Ti_3_C_2_T_x_ materials (NM_2_P_1_) exhibited a capacitance of 630 F g^−1^ 10 A cm^−3^ under light, representing a 35.9% improvement over dark conditions. Detailed mechanistic studies revealed that during photo‐assisted charging, NCDs generated photogenerated electron pairs where the electrons transferred efficiently to the Ti_3_C_2_T_x_ matrix, promoting increased cation accumulation between the MXene interlayer gaps (**Figure** **13**a). This electron transfer is likely facilitated by N‐doped defects in NCDs, which modify the local electronic structure to lower charge transfer barriers, analogous to defects in semiconductors enhancing interfacial charge transport in related systems. According to the TPV test, NCDs enhanced the light absorption capacity, increasing the photo‐induced charge generation (Figure 13b) and improving the charge transfer kinetics of the NM_2_P_1_ composite electrodes (Figure 13c).

Chang et al. developed nanohybrid materials combining CDs with 2D Ti_3_C_2_T_x_ MXene to improve the pseudocapacitance driven by photothermal effects.^[^ ^30^ ^]^ Under solar illumination, the photothermal electrode could be heated locally, which effectively solved the problem of EC power attenuation at low temperatures without affecting the temperature of the electrolyte. The introduced homogeneous distribution of CDs on the surface of MXene helped inhibit the self‐restacking of MXene. They also benefited from uniform electrode heating (Figure 13d) and fast charge transfer (Figure 13e). The photothermal ECs revealed gradually increased capacitance performance with increasing solar power density (Figure 13f–h).

The application of CDs in photo‐assisted ECs, although still in its infancy, has demonstrated significant potential through performance enhancements such as increased capacitance under illumination and improved charge transfer kinetics. These advancements stem from CDs' unique ability to bridge light absorption, charge generation, and electrochemical storage—properties that position them as promising candidates for next‐generation solar‐driven energy storage.

However, mechanistic understanding remains relatively limited, particularly regarding the underlying relation between CD features and the electrochemical performance of ECs under light irradiation. For instance, the intricate relationship between CD surface defects (e.g., heteroatom dopant sites, oxygen vacancies), charge carrier separation efficiency, and the resultant capacitance enhancement under illumination remains poorly understood, lacking rigorous mechanistic dissection. Current studies generally highlight these phenomena collectively, but the relative contributions of individual processes (such as whether capacitance gains arise primarily from photogenerated charge carriers, photothermal effects, or synergistic interactions) remain ambiguous.

That gap partly stems from the limited studies in this emerging field, where research has predominantly focused on phenomenological observations and performance optimization rather than mechanistic elucidation. Consequently, it is critical to integrate advanced characterization techniques (e.g., time‐resolved spectro‐electrochemistry) with targeted experiments to reveal the underlying interaction mechanisms. These approaches can clarify how CDs’ structure (e.g., sp^2^ domain size, surface functional groups) tunes charge separation efficiency and interfacial transfer under light. Furthermore, theoretical calculations, such as DFT calculations to model charge delocalization at CDs‐electrode interfaces can further validate and refine these mechanistic insights. Such efforts will not only clarify the underlying mechanisms but also guide the rational design of CDs‐based photo‐assisted ECs, bridging the current gap between experimental observations and fundamental understanding.

**Table 2:** The representative performance data of photo‐assisted ECs based on CDs.

Photoactive material	Device configuration	Light condition	Capacitance under light	Enhancement vs dark	Refs.
OPC‐CDs‐700	Symmetric EC (H_2_SO_4_ electrolyte)	400–800 nm (150 mW cm^−2^)	312 F g^−1^ @ 0.1 A g^−1^	54.4%	[28]
CDs/ZnO/SWCNT	Hybrid EC (KOH electrolyte)	UV light (100 mW cm^−2^)	1.53 mF cm^−2^ at 1.25 µA cm^−2^	—	[178]
CDs/thylakoids (Thy)	Zn‐ion hybrid capacitor	Light (15 mW cm^−2^)	80 mAh g^−1^	144%	[32]
RCDs@Ni‐MoS_2_	Symmetric EC (PVA gel)	350 W Xenon light (120 mW cm^−2^)	7.04 mF cm^−2^	94.57%	[33]
NCDs/Ti_3_C_2_T_x_ MXene	Fiber EC (H_2_SO_4_/PVA gel)	400–800 nm (150 mW cm^−2^)	630 F g^−1^	35.9%	[179]
CDs/MXene	Symmetric EC (H_2_SO_4_ gel)	Solar simulation (223.58 mW cm^−2^)	10.47 F cm^−2^	304%	[30]

## 6 Conclusions and Outlook

This review systematically discusses the unique properties of multifunctional CDs and their research advances in ECs. Due to the diverse charge storage mechanisms arising from the various configurations of ECs, the regulation of electrodes and electrolytes is crucial to achieving high‐performance ECs. The introduction of CDs to ECs as additives for electrode materials and electrolytes demonstrates significantly enhanced electrochemical performance. The fabrication of CDs presents significant advantages, including broad raw material sources, flexible synthesis methods, and environmental friendliness, which provides a good opportunity for ECs. CDs possess unique structural features, including nanoscale size (<10 nm), high specific surface area, porous architecture, and tunable surface functional groups. These properties enhance both electric double‐layer capacitance and pseudocapacitance.

When used as an electrode additive, small‐sized CDs can tailor the surface structures and morphology of composite materials, forming available nanoporous channels and increasing the surface area that improves the electrochemical performance of ECs. The surface of CDs with abundant functional groups enhances electrolyte wettability, which benefits the effective penetration of electrolytes into the electrode. The good electrical conductivity of CDs considerably reduces the interface resistance and facilitates charge transfer kinetics, thus enhancing the capacitive performance of ECs. Additionally, CDs as electrolyte additives increase the ionic conductivity of electrolytes and improve the affinity of the electrode, boosting the capacitance performance of ECs.

Regarding the photoelectric properties, CDs exhibit broad‐spectrum light absorption, efficient separation of photogenerated carriers, and photothermal conversion effects. These characteristics are particularly critical for photo‐assisted ECs. Benefiting from their unique size effects, tunable surface structures, and rapid photoresponse, CDs offer novel pathways to overcome the limitations of traditional ECs. Photo‐assisted ECs demonstrate especially, promising potential for self‐powered operation and high energy density.

Although CD‐based ECs have many advantages, there are still several challenges:

1. A deep understanding of the fine tailoring of CD structures is needed. Preparing CDs with uniform sizes and controllable surface structures is still challenging. The size and morphology of CDs significantly affect their specific surface area and ion transport pathways. Current synthesis methods (e.g., pyrolysis and hydrothermal methods) fail to achieve precise regulation on CD size, crystallinity, and surface functional groups, leading to poor batch reproducibility. Additionally, heteroatom doping and functional group modification of CDs remain challenging to achieve precise modulation, which directly influences pseudocapacitance contributions and electrolyte compatibility in ECs. The excessive doping in CDs can destroy the graphite structure, decreasing conductivity. The surface functional groups, such as the hydroxyl group, carboxyl group, and nitrogen‐containing functional group, can enhance the water solubility of CDs and their affinity with other materials. However, excessive surface functional groups will also reduce the capacitance of composite materials. Therefore, the practical applications of CDs in ECs require developing fabrication technologies to precisely tune the structures of CDs.
2. Much more effort should be made to reveal the underlying reaction mechanisms of CD‐based ECs through the integration of advanced in situ characterization and theoretical modeling. The complexity of CDs’ structures (carbon cores with rich surface groups) complicates understanding of charge storage mechanisms: high specific surface area and porosity influence electric double‐layer capacitance, while surface functional groups regulate redox kinetics for pseudocapacitive contributions. To clarify this issue, techniques such as in situ Raman spectroscopy can be used to track real‐time structural changes during cycling, and liquid cell in situ transmission electron microscopy (in situ TEM) can directly observe the behavior of CDs during charge‐discharge cycles, monitoring whether they aggregate, dissolve, or detach from the electrode matrix (all of which are key factors leading to capacitance decay). Combining these with DFT calculations to map charge transfer pathways will clarify how CDs synergistically enhance capacitance and stability, guiding rational EC design.
3. CDs for photo‐assisted ECs should be further investigated. Current research is in its infancy. The principal challenge is efficient photo‐generated carrier separation. Avoiding the recombination of photo‐generated electrons and holes is critical for improving photoelectric conversion efficiency. Second, the interaction mechanisms between surface functional groups of CDs and electrolyte ions under light irradiation still lack understanding, which impedes the quantification of pseudocapacitance contributions. Moreover, the structural stability of CDs under light irradiation should be considered. Long‐term light exposure triggers photo‐oxidative decomposition of surface functional groups on CDs, leading to pseudocapacitance decay and reduced cycling stability of ECs. In this regard, standardized light/dark cycling tests should be established, and research should be conducted in combination with in situ characterization and theoretical modeling under the synergistic effect of light irradiation and applied voltage. Finally, the integration and scalability of photo‐assisted ECs face bottlenecks. The design of integrated structures is key to satisfying requirements for light absorption and charge storage.
4. The cost of practical application of CDs‐based ECs is another key challenge that needs to be considered from multiple dimensions. The synthesis of CDs has duality: on the one hand, raw materials are widely available and low‐cost, which significantly reduces material costs; on the other hand, the relatively complex preparation process may increase costs. However, the performance advantages of CDs can offset the synthesis costs. Although their preparation costs may be higher than those of traditional carbon materials such as activated carbon, CDs have excellent properties such as high specific surface area, adjustable surface functional groups, and excellent conductivity, which enable ECs to obtain higher specific capacitance and cycling stability, thus having a competitive overall cost‐performance ratio. Compared with advanced materials such as graphene (which requires complex redox processes) and MXenes (which rely on harmful fluoride etching), CDs rely on low‐cost precursors and scalable synthesis routes to reduce raw material costs while achieving comparable or even better performance. It is also crucial to ensure long‐term cycling stability, as CDs‐based ECs need to meet industry standards at least 10000 cycles (with 80% capacitance retention) for consumer electronics and at least 50000 cycles for grid‐scale applications. That requires improving electrode preparation processes to enhance the combination of CDs with the electrode matrix and developing advanced electrolytes with improved compatibility for the CD surfaces.

## Conflict of Interest

The authors declare no conflict of interest.
